# Estimating State-Specific Contributions to PM_2.5_- and O_3_-Related Health Burden from Residential Combustion and Electricity Generating Unit Emissions in the United States

**DOI:** 10.1289/EHP550

**Published:** 2016-09-02

**Authors:** Stefani L. Penn, Saravanan Arunachalam, Matthew Woody, Wendy Heiger-Bernays, Yorghos Tripodis, Jonathan I. Levy

**Affiliations:** 1 Department of Environmental Health, Boston University School of Public Health, Boston, Massachusetts, USA; 2 Institute for the Environment, University of North Carolina at Chapel Hill, Chapel Hill, North Carolina, USA; 3 U.S. Environmental Protection Agency, Durham, North Carolina, USA; 4 Department of Biostatistics, Boston University School of Public Health, Boston, Massachusetts, USA

## Abstract

**Background:**

Residential combustion (RC) and electricity generating unit (EGU) emissions adversely impact air quality and human health by increasing ambient concentrations of fine particulate matter (PM_2.5_) and ozone (O_3_). Studies to date have not isolated contributing emissions by state of origin (source-state), which is necessary for policy makers to determine efficient strategies to decrease health impacts.

**Objectives:**

In this study, we aimed to estimate health impacts (premature mortalities) attributable to PM_2.5_ and O_3_ from RC and EGU emissions by precursor species, source sector, and source-state in the continental United States for 2005.

**Methods:**

We used the Community Multiscale Air Quality model employing the decoupled direct method to quantify changes in air quality and epidemiological evidence to determine concentration–response functions to calculate associated health impacts.

**Results:**

We estimated 21,000 premature mortalities per year from EGU emissions, driven by sulfur dioxide emissions forming PM_2.5_. More than half of EGU health impacts are attributable to emissions from eight states with significant coal combustion and large downwind populations. We estimate 10,000 premature mortalities per year from RC emissions, driven by primary PM_2.5_ emissions. States with large populations and significant residential wood combustion dominate RC health impacts. Annual mortality risk per thousand tons of precursor emissions (health damage functions) varied significantly across source-states for both source sectors and all precursor pollutants.

**Conclusions:**

Our findings reinforce the importance of pollutant-specific, location-specific, and source-specific models of health impacts in design of health-risk minimizing emissions control policies.

**Citation:**

Penn SL, Arunachalam S, Woody M, Heiger-Bernays W, Tripodis Y, Levy JI. 2017. Estimating state-specific contributions to PM_2.5_- and O_3_-related health burden from residential combustion and electricity generating unit emissions in the United States. Environ Health Perspect 125:324–332; http://dx.doi.org/10.1289/EHP550

## Introduction

Elevated concentrations of ambient ozone (O_3_) and fine particulate matter ≤ 2.5 μm in aerodynamic diameter (PM_2.5_) contribute to adverse health outcomes in exposed populations (Jerrett et al. 2009; Krewski et al. 2009). Epidemiological literature has described relationships between population exposure to these air pollutants and chronic and acute health effects, including premature mortality (Brook et al. 2002; Bell et al. 2004; Ito et al. 2005; Levy et al. 2005) and multiple morbidities (Zanobetti et al. 2009; Ji et al. 2011; Mustafic et al. 2012; Levy et al. 2012).

A number of emitting source sectors that are spatially distributed across the United States contribute to total ambient concentrations of these pollutants, including electricity generating units (EGUs), which burn fossil fuels like coal and natural gas to produce electricity, and residential combustion (RC) sources, including oil and natural gas-burning furnaces or wood-burning stoves to heat homes. Among the most significant contributors to air pollution-related health impacts are emissions related to EGUs, which are elevated stack point sources, and area sources, which are ground-level, widely distributed sources and include RC. In 2005, Fann et al. (2013) estimated EGUs contribute 38,000 premature deaths per year across the United States, highest among source sectors, with area sources contributing another 27,000 premature deaths per year. Similarly, a recent study estimated that EGUs contributed 53,900 premature deaths from PM_2.5_ and O_3_ across the United States in 2005, while commercial and residential combustion together contributed 42,150 deaths from PM_2.5_ and O_3_ (Caiazzo et al. 2013). Another recent study estimated PM_2.5_-related health risks of 41,660 premature deaths from EGUs and 35,790 premature deaths from commercial and residential combustion (Dedoussi and Barrett 2014).

While these comparisons provide valuable insight about high-priority source sectors, they do not include information on impacts of specific emitted pollutants from individual states and source types for both PM_2.5_ and O_3_. Caiazzo et al. (2013) estimated total premature mortalities by state from a receptor perspective rather than a source perspective (i.e., the premature mortalities for populations living in California rather than the premature mortalities attributable to sources in California) and do not differentiate by emitted pollutant, providing less useful information from a control strategy perspective. Dedoussi and Barrett (2014) estimate total premature mortalities by source-state for PM_2.5_ using a different modeling approach [adjoint modeling using GEOS (Goddard Earth Observing System)–Chem chemical transport model (http://www.geos-chem.org) with slightly coarser resolution] and lacking insight about O_3_-related impacts or impacts by source-state and precursor pollutant. GEOS-Chem chemical transport model with KPP chemical solver and RPMARES aerosol equilibrium model were used. Many federal policies targeting EGUs, including the U.S. Environmental Protection Agency’s (EPA) Clean Power Plan (U.S. EPA 2015a) and Cross-State Air Pollution Rule (U.S. EPA 2016) have mechanisms for differential actions by states, and it is important to understand how alternative combinations of emissions reductions could influence public health. RC may be influenced by a policy like the Clean Power Plan, which considers energy efficiency as one mechanism to achieve emissions reductions, and may be directly targeted as part of State Implementation Plans (SIPs) or other state policy measures. Quantification of source-specific and pollutant-specific health risks by source-state provides a tool for policy makers to create efficient emission control strategies.

Premature mortalities from different source sectors can be estimated by combining source-specific air quality changes with population characteristics and epidemiologically derived concentration–response functions (Fann and Risley 2013; Hubbell et al. 2009; Tagaris et al. 2009). In addition to determining total premature mortalities, health damage functions (estimated as premature mortalities per unit emissions) can be calculated to provide insight about sources and locations in which emissions reductions are more or less efficient from a public health perspective. Heterogeneity in health damage functions is associated with ambient atmospheric chemistry and meteorology, source and chemical profiles of emitted pollutant precursors, and the geographic distribution of exposed populations (Fann et al. 2009). EGU and RC sources provide interesting contrasts: EGUs are individual point sources that vary in location, stack height, age, and efficiency, while RC is a ground-level area source spread over a wider area and directly tied to population patterns. Both are spatially distributed across the United States, with between-sector and within-sector differences including proximity to populations, height of emissions origin, and atmospheric chemistry and meteorology in each location and downwind. Analysis of RC and EGUs specifically allows us to consider two sectors that would be influenced by policies such as the Clean Power Plan that target EGUs but could have ancillary effects on RC (e.g., through residential energy efficiency).

The Community Multiscale Air Quality (CMAQ) model, a peer-reviewed atmospheric chemistry and transport model capable of modeling gas-phase, aerosol, and aqueous chemistry including the formation of O_3_ and PM_2.5_ from emitted precursors, can predict changes in ambient air quality associated with these two source sectors, among others (Byun and Ching 1999; Byun and Schere 2006). Utilized with the decoupled direct method (DDM), which decouples sensitivity equations from model equations to allow for stability and accuracy of values and computational efficiency, CMAQ-DDM has the power to determine individual source contributions by analyzing the sensitivity of ambient concentrations of PM_2.5_ and O_3_ to specific precursor emissions in the presence of different atmospheric and meteorological conditions (Dunker 1984; Dunker et al. 2002; Koo et al. 2007). CMAQ-DDM has been used in previous studies to quantify exposure to pollutants from source-tagged precursors (Bergin et al. 2008; Odman et al. 2002; Itahashi et al. 2012), and has been used to assess health impacts due to climate change in the United States (Tagaris et al. 2010).

In this study, we quantified premature mortalities from EGUs and RC for each emitted pollutant and source-state individually across the continental United States. We used CMAQ version 4.7.1 (Byun and Ching 1999; Byun and Schere 2006) instrumented with DDM-3D (Dunker 1984; Napelenok et al. 2006) to determine estimated changes in ambient pollutant concentrations of PM_2.5_ and O_3_ based on EGU and RC emissions, using these air quality changes to determine predicted total premature mortalities and health damage functions by source-state and sector. This approach will allow state and federal policy makers to determine which sources to target to decrease public health burdens and which policies will be most efficient in achieving improvements. Comparisons of health damage functions by source sector and source-state will allow further assessment of differential attributes of RC and EGU emissions.

## Methods

### Study Design

**Figure 1:**
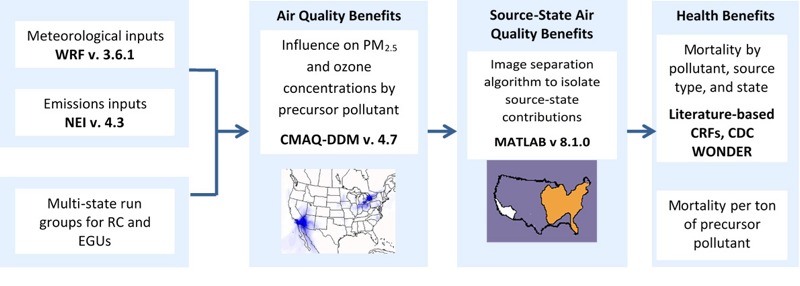
Health damage function model inputs and outputs.

Key model components are presented in Figure 1. Briefly, to determine changes in ambient air quality associated with EGUs and RC, we used CMAQ (Byun and Ching 1999; Byun and Schere 2006) instrumented with DDM in three dimensions (Dunker 1984; Napelenok et al. 2006). This model isolated PM_2.5_- and O_3_-specific contributions from state-wide EGU and RC precursors to assess the sensitivity of ambient pollutant concentrations to these precursors. Resultant ambient pollutant concentrations were then linked with population and mortality rate data from the Centers for Disease Control and Prevention (CDC 2015). Concentration-response functions associating ambient pollutant concentrations with health effects were derived from the epidemiological literature. We estimated total premature mortalities for each source sector by source-state for each precursor pollutant-ambient concentration relationship, including primary elemental carbon (PEC), primary organic carbon (POC), and primary sulfate (PSO_4_) as primary PM_2.5_ precursors; nitrogen oxides (NO_x_), sulfur dioxide (SO_2_), and volatile organic compounds (VOCs) as secondary PM_2.5_ precursors; and NO_x_ and VOCs as O_3_ precursors, detailed in Table S1. We also estimated health damage functions, or premature mortality risk per 1,000 tons of precursor emissions. Emissions details can be found in Figures S1 and S2 and Tables S2 and S3.

### CMAQ-DDM Design and Modeling

Due to the computationally intensive nature of CMAQ-DDM, it was not practical to construct separate runs for each source sector and source-state. To maximize efficiency, we incorporated one to three states into a single DDM run for each of RC and EGUs, and we developed algorithms to separate the concentration impacts from each state (described in Section 2.3 and in “Image Segmentation Algorithm” in the Supplemental Material). To design these runs, we overlaid concentration surface results from a pilot analysis of SO_2_ tracer emissions from multiple source-states and grouped states to minimize errors in source-state attribution with the smallest number of runs. For EGUs, a subset of states cut across electricity dispatch regions, so we subdivided those states into two areas to facilitate future connection with energy efficiency or renewable energy projects. In total, 65 model runs were conducted (described in Table S4), including 25 groups of states for modeling RC and 40 groups of states (and partial states) for modeling EGUs.

Details of the CMAQ-DDM modeling are provided in “Community Multiscale Air Quality (CMAQ) model” in the Supplemental Material. RC sources were modeled as low-level area sources including all residential fuel types, aggregated to county level for apportionment to grid cells by state. EGUs were modeled by power plant and aggregated to grid cells by state. Cells of 36 km × 36 km covering the continental United States were used to grid state-specific emissions from each source sector. Because modeling the full year was computationally intensive, we selected 2 months (January and July) to provide bi-seasonal representation, using all-source emissions and meteorology from 2005. To provide initial background conditions, a spin-up period of 11 days prior to each month was simulated. Whole-month sensitivity values from January and July were averaged to represent annual estimated contributions of statewide RC and EGU sources to ambient PM_2.5_ and O_3_ concentrations. Values are reported as 24-hr averages for PM_2.5_ constituents and 8-hr maximum values for O_3_ for consistency with current regulatory policies. These values were used in total health impact and health damage function calculations.

### Separation of State-Specific Concentration Surfaces

To separate contributions of individual source-state’s contributions to ambient concentrations from one another within a DDM run, we applied image separation techniques using MATLAB 8.1.0, R2013a (MathWorks, Natick, MA). We developed a region-growing algorithm to determine regions of concentrations attributable to each source-state for each emitted precursor and associated ambient pollutant relationship within each model run and season. This algorithm allowed for both positive and negative sensitivities to be included within regions, and ensured that within a run, a smaller state’s region could capture the extent of its health impacts. Quality assurance (QA) analyses were performed, including analysis of total health impact and health damage function distributions for resultant health values, as well as visual inspection of concentration surfaces. For runs that did not meet QA criteria, we re-ran CMAQ-DDM for individual states in isolation. This process allowed determination of emissions impacts from individual source-states within a CMAQ-run group. “Image Segmentation Algorithm” in the accompanying Supplemental Material contains more information regarding the image segmentation algorithm.

### Total Health Impact Calculation and Health Damage Function Modeling

Calculation of total premature mortalities by source-state and source sector is analogous to the calculation of health damage functions, with the exception of normalization by precursor emissions. Changes in air quality associated with state-wide emissions were linked with premature deaths using a standard health impact modeling equation, calculated separately for each precursor and associated ambient pollutant pair for each source sector. The equation is as follows:


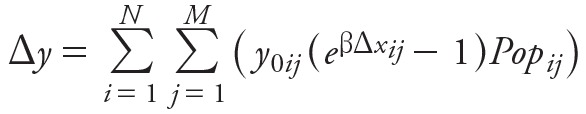


where *i* is row number and *j* is column number, *N* is total number of rows and *M* is total number of columns in the CMAQ grid. Δ*y* is change in mortality across the continental United States, *y*_0_ is baseline mortality incidence rate in grid cell at location *ij*, β is concentration–response function as derived from the epidemiological literature, Δ*x* is change in air quality for a given precursor in grid cell *ij*, and *Pop* is the population of interest in grid cell *ij*. To associate premature mortalities with PM_2.5_ concentrations, we applied a central estimate concentration-response function of a 1% increase in mortality associated with every 1-μg/m^3^ increase in annual average PM_2.5_ concentration (Roman et al. 2008). To associate premature mortalities with O_3_ concentrations, we applied a central estimate concentration–response function of 0.4% increase in daily mortality per 10-ppb increase in daily 8-hr maximum O_3_ concentrations, based on major multi-city and meta-analysis studies that evaluated health impacts across the year (Ji et al. 2011; Bell et al. 2004, 2005; Ito et al. 2005; Levy et al. 2005; Schwartz 2005). To estimate county-wide population and baseline mortality rates for adults ≥ 25 years old in 2005, values from 2001 to 2010 were obtained from CDC WONDER (CDC 2015) and averaged for stability of values. County-wide values were projected as Lambert conformal conic in ArcMap (version 10.1; ESRI, Redlands, CA, USA) and intersected with grid cells, assuming uniform density of population and mortality rate within counties.

Total premature mortalities were calculated by emitted precursor and associated ambient pollutant pair for each source-state for both EGUs and RC, assuming January and July each represent 6 months. These 6-month values were summed to obtain annual health impact estimates. Health damage function values were calculated by normalizing total premature mortalities by total amount of emitted precursor for January and July, each representative of what the health damage function would be if these individual month conditions were present for an entire year. Annual health damage function estimates were calculated by averaging January and July health damage functions, interpreted as the mortality risk associated with uniform emissions across the year. Ozone estimates were calculated for both January and July given epidemiological evidence based on year-round exposures.

### Comparison of RC and EGU Source Sectors

Descriptive statistics were calculated for total premature mortalities and health damage functions for EGU and RC by precursor and source-state. We examined between-state variation in total premature mortalities and health damage functions by source sector and precursor pollutant, as well as between-pollutant and within-state variation. To facilitate interpretation, we calculated the percentage of source-state mortalities found within that state (i.e., percentage of deaths from California RC emissions that occur in California), examined emissions inventories and mapped source locations.

## Results

### Total Health Impacts

**Figure 2:**
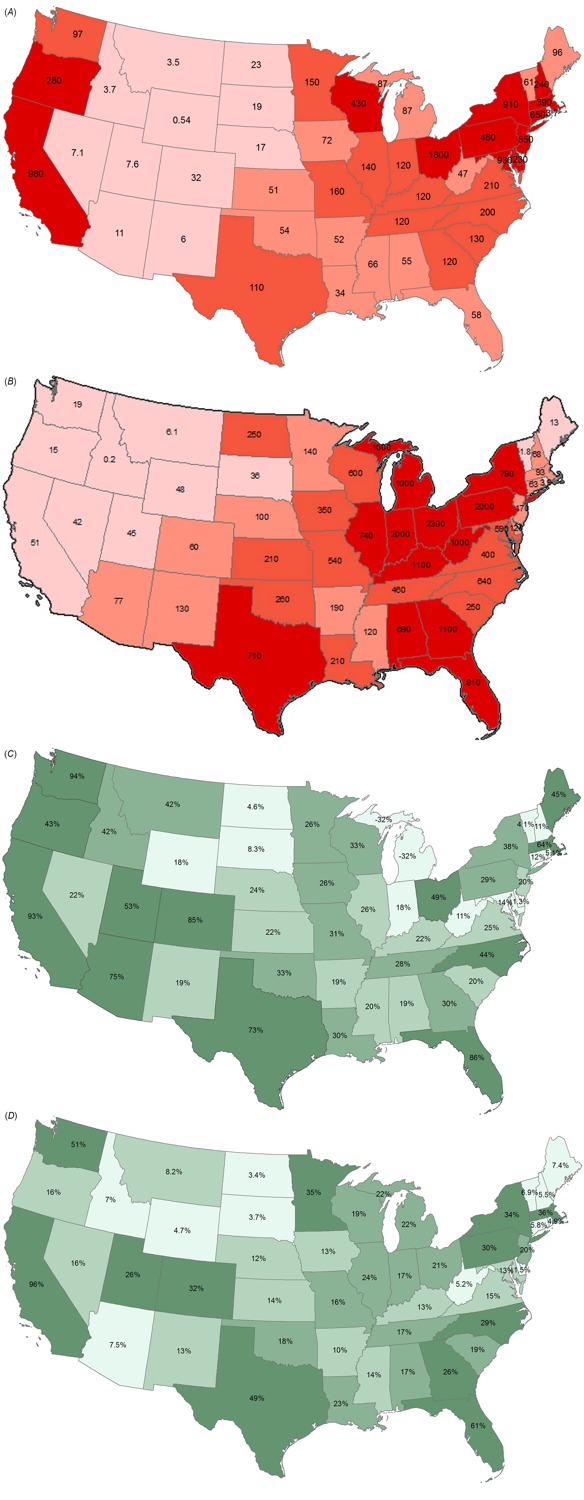
(*A*) Total premature deaths associated with source-state RC emissions (e.g., California RC emissions caused 980 premature deaths across all states). (*B*) Total premature deaths associated with source-state EGU emissions (e.g., EGU emissions from Ohio caused 2,300 premature deaths across all states) (U.S. Census Bureau 2016). (*C*) Percentage of source-state premature deaths from RC emissions occurring in the source-state (e.g., 93% of the 980 premature deaths from California RC emissions occurred in California). (*D*) Percentage of source-state premature deaths from EGU emissions occurring in the source-state (e.g., 21% of the 2,300 premature deaths from Ohio EGU emissions occurred in Ohio).

Total number of premature mortalities per year for each precursor were modeled for each state for both RC and EGUs (Figure 2; see also Tables S5 and S6). RC contributes 10,000 additional deaths per year, and EGUs contribute 21,000 additional deaths per year from both PM_2.5_ and O_3_.

States contributing the most deaths related to RC are those with combustion-type home heating near or upwind of highly populated areas, including Ohio, California, Maryland, and New York (Figure 2a). RC emissions are tied to population, so highly populated areas will have both greater emissions and greater exposed populations. Primary PM_2.5_ precursors contribute 74% of premature mortalities for RC, driven by POC, and the vast majority of primary PM_2.5_ emissions are associated with wood burning (see Figure S2). The percentage of RC-related premature mortalities found within the source-state varies widely across states (Figure 2C), with values exceeding 75% in geographically large states without substantial downwind populations (e.g., Washington, California, Florida) and values below 10% in smaller states with large downwind populations (e.g., Washington District of Columbia, Delaware, Vermont).

States with the greatest total mortalities from EGUs are those with the greatest coal-fired power plant emissions upwind of highly populated areas, including Ohio, Indiana, and Pennsylvania (Figure 2B). For EGUs, SO_2_ contributes most to premature mortality burden, with 77% of premature mortalities related to secondary PM_2.5_ or O_3_ attributable to SO_2_ and NO_x_. The vast majority of SO_2_ and NO_x_ emissions from EGUs are related to coal combustion (see Figure S1). As anticipated, given the dominance of secondarily-formed pollutants, the percentage of premature mortalities found within the source-state for EGUs is less than that for RC (22% vs. 38% overall). In contrast to RC, only 3 states have more than half of their EGU-related health impacts within the source-state (California, Florida, and Washington), with only 12 states having > 25% of their EGU-related health impacts within the source-state (Figure 2D).

Ratios of RC-related deaths to EGU-related deaths vary greatly across source-states. Deaths from RC exceed those from EGUs for source-states in the Northeast and West Coast where population density is high, EGU coal combustion is limited, and wood or oil is used in some homes for heating. In contrast, deaths from EGUs exceed those from RC in source-states with appreciable EGU coal combustion and significant usage of electricity for home heating. Excluding the five lowest emitting states for primary PM_2.5_ from RC and SO_2_ from EGUs, where health damage functions may be biased due to limited emissions (explained further in Section 3.2), ratios of EGU-related deaths to RC-related deaths vary from 0.05 to 20 across source-states.

There is significant seasonal variation in total premature mortalities by source sector and precursor-pollutant pair. RC-related deaths are dominated by cold weather emissions, as deaths are 20 times greater for January (representing cold months) versus July (representing warm months). RC emissions are greatest for January in the Northwest, Midwest and Northeast, driven by climate, population density, and fuel types (see Figure S3). Conversely, EGU-related deaths are 5 times greater for July than for January, given the substantial contribution from SO_2_ emissions and enhanced secondary particle formation from SO_2_ in warmer seasons. EGU emissions of SO_2_ are most prominent in the Midwest and Mid-Atlantic regions (see Figure S4). The impact of NO_x_ on O_3_ has an inverse relationship with deaths in January due to O_3_ titration in cold weather and a positive relationship with deaths in July, as high temperatures are needed for O_3_ formation and high ambient NO_x_ can contribute to VOC-limited regimes.

### Health Damage Functions

**Figure 3:**
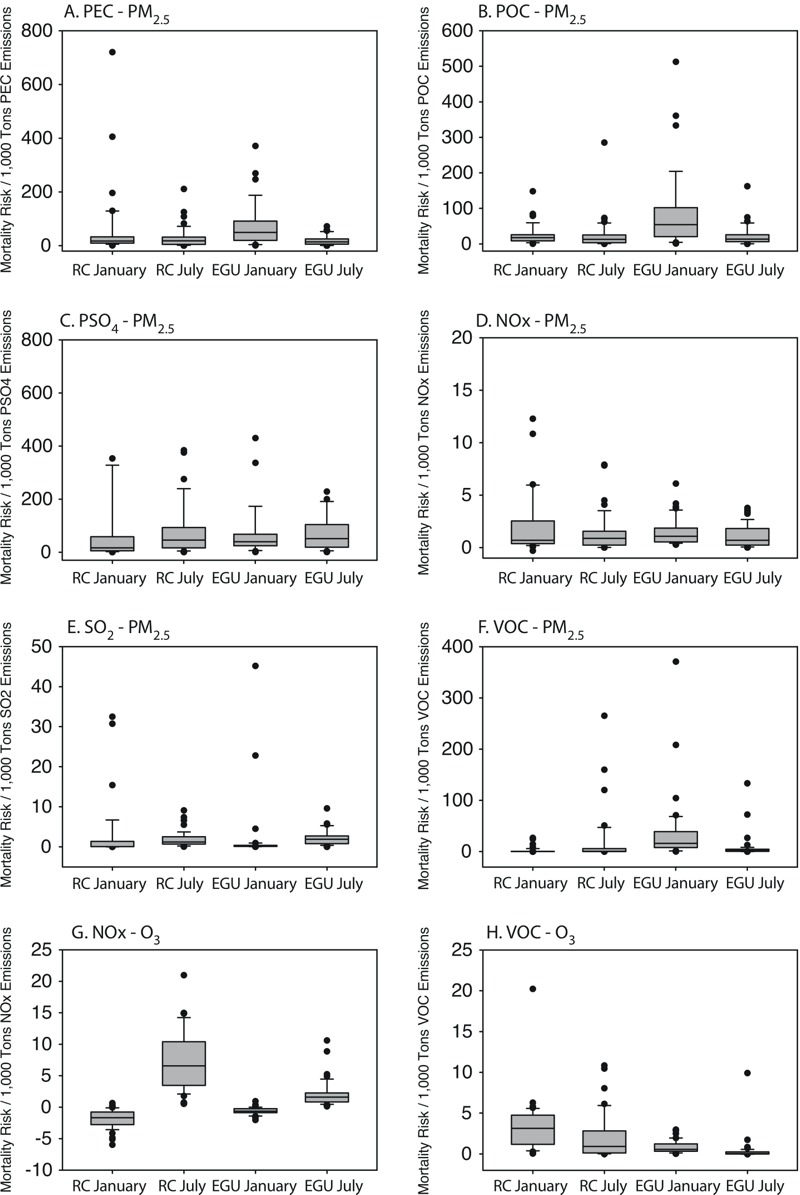
Box plots of health damage functions for RC and EGUs for January and July by precursor-pollutant pair. (*A*) Health damage functions as mortality risk per 1,000 tons precursor emissions for PM_2.5_ related to PEC; (*B*) PM_2.5_ related to POC; (*C*) PM_2.5_ related to PSO_4_; (*D*) PM_2.5_ related to NO_x_; (*E*) PM_2.5_ related to SO_2_; (*F*) PM_2.5_ related to VOC; (*G*) O_3_ related to NO_x_; (*H*) O_3_ related to VOC. Note: *y*-axes display different ranges for each panel. Boxplots show 5%, first quartile, median, third quartile, and 95% values for each precursor and associated pollutant damage function.

Health damage functions for RC and EGUs were modeled for each precursor and season for each source-state. Figure 3 shows the distribution of health damage functions for RC and EGUs by precursor for January and July. Health damage functions for primary PM_2.5_ precursors are greatest on average for January EGU emissions, while distributions of RC and EGU July health damage functions for primary PM_2.5_ precursors are similar to one another. States with very low emissions provide abnormally inflated health damage functions, which have been excluded from Figure 3 (but shown in Tables S7–S10).

Across both source sectors, health damage function values are much smaller for secondary pollutants compared with primary pollutants. SO_2_-PM_2.5_ damage functions display more seasonality than NO_x_-PM_2.5_, with heightened impacts per unit emissions in July. NO_x_-O_3_ health damage functions are generally negative for RC in January but positive in July. NO_x_-O_3_ health damage functions for EGUs display smaller negative values in January and less variability overall. VOC-O_3_ health damage functions are significantly higher for RC than for EGUs in both seasons.

While Figure 3 is able to show the range of health damage functions for both source sectors, it does not describe their relationship on a state-by-state basis, which is important in understanding relative magnitudes of pollutant impacts from different sources. The relationship between health damage functions for RC and EGUs varies greatly across states (see Figures S5 and S6). Many states with low RC primary PM_2.5_ health damage functions also have low EGU primary PM_2.5_ damage functions, especially for July emissions. States where RC and EGU primary PM_2.5_ health damage functions differ greatly from one another (e.g., South Dakota, Montana, Maine, Oklahoma) tend to be large low-population states where EGUs are located in areas geographically removed from the locations of RC combustion (see Figures S3 and S4). In comparison with primary PM_2.5_, the association between EGU and RC health damage functions is similar for NO_x_ but not SO_2_ and VOCs. O_3_-related health damage functions for EGUs are smaller in magnitude than those for RC, with an inverse association between RC and EGU values.

## Discussion

We estimated the premature mortality burden of ambient PM_2.5_ and O_3_ concentrations attributable to RC and EGU emissions by source-state and precursor pollutant in the continental United States using CMAQ-DDM and health damage function modeling based on 2005 air quality and population estimates. Health impacts of these source sectors have not previously been compared directly, nor has the literature provided insight about dominant pollutants and source-states. We quantify 10,000 additional premature deaths per year due to RC emissions and 21,000 additional premature deaths per year due to EGU emissions, with RC health impacts dominated by PEC and POC emissions and EGU health impacts dominated by SO_2_ and NO_x_ emissions (forming PM_2.5_ and O_3_).

### Comparing Total Health Impacts with Other Studies

While comparisons with previous studies are challenging given underlying model differences, examination of similarities and differences in estimates can provide insights about our findings. Total mortalities associated with EGUs have been previously calculated for the continental United States for 2005 from PM_2.5_ and O_3_ (Caiazzo et al. 2013; Fann et al. 2013). Fann et al. (2013) found EGUs were responsible for 38,000 premature deaths in 2005 versus the 21,000 in our study. For RC, while Fann et al. (2013) do not report a value directly, their sectoral values imply approximately 8,000 deaths per year from residential wood combustion. The vast majority of our 10,000 attributable premature deaths are likely related to wood combustion given its dominance in primary PM_2.5_ emissions. In addition, EPA recently published a regulatory impact analysis for residential wood heaters and utilized data from Fann et al. (2013) to determine 0.07 deaths per ton of primary PM_2.5_ emissions (U.S. EPA 2015b), identical to our national average value. Caiazzo et al. (2013) estimated EGUs caused 52,000 premature deaths from PM_2.5_ and 1,700 premature deaths from O_3_, and commercial and residential combustion combined contributed 41,800 deaths from PM_2.5_ and 350 deaths from O_3_ in 2005. While we found O_3_ contributed 2,000 premature deaths from EGUs and 320 premature deaths from RC, values in line with Caiazzo et al. (2013) estimates, our estimates for PM_2.5_-related premature deaths are a factor of 2–3 lower for EGUs and a factor of 4 lower for RC, albeit with commercial combustion included in Caiazzo et al. (2013). All three studies analyzed health impacts for 2005 conditions using the National Emissions Inventory, yet magnitude differences are expected given utilization of different atmospheric dispersion models [CMAQ-DDM, version 4.7.1 in our study; CMAQ, version 4.7.1 brute force in Caiazzo et al. (2013), Comprehensive Air Quality Model with Extensions (CAMX), version 5.30 using SMAT/MATS for Fann et al. (2013)] and different concentration–response functions.

### Total Health Impact Analysis

Total health impacts from RC are driven by POC emissions across the United States. The number of deaths caused by each source-state is related to population, which influences both the extent of residential emissions and size of the exposed population, the need for home heating, and the degree to which wood, oil, and gas are used. As such, states causing the most deaths from RC have large populations within the state and immediately downwind and experience cold weather. In contrast, while downwind population plays a role for EGU-related premature mortalities, SO_2_ emissions patterns from EGUs differ greatly from POC emissions patterns from RC, and regional-scale atmospheric chemistry and transport plays a more significant role. States with the greatest EGU health impacts have the greatest coal-fired power plant emissions and atmospheric conditions amenable to secondary PM_2.5_ formation, specifically sulfate aerosol that is abundant in the eastern United States (Bell et al. 2007) during summer months. Our analyses of geographic patterns of health impacts reinforces the greater spatial extent of impact for secondarily formed pollutants from EGUs versus primarily emitted pollutants from RC.

### Health Damage Function Analysis

Health damage functions do not follow the same patterns as total health impacts. Considering between-state differences, states with high health damage functions for primary PM_2.5_ emissions are similar for RC and EGUs, largely in the Northeast and Mid-Atlantic regions. The highest health damage functions for secondary PM_2.5_ precursors are in those same regions, with higher population states having higher health damage functions for RC than for EGUs. Western states, which tend to have lower populations with other low population states surrounding them, have the lowest health damage functions for primary PM_2.5_ precursors, but not secondary PM_2.5_ precursors, as they may be in areas that favor secondary particulate formation. O_3_-related health damage functions follow different patterns, with a tight association between values for EGUs and RC for both NOx and VOCs.

### Limitations

Despite this study’s use of a sophisticated air quality model and epidemiologically derived concentration–response functions to estimate total premature mortalities and health damage functions associated with RC and EGU emissions, there are a number of limitations, some of which are related to computational limitations. To determine sensitivity of ambient pollutant concentrations to precursor emissions from a source it is advantageous to model each source individually for an entire year. Due to computational constraints we chose not to model each state’s emissions individually and instead created CMAQ-DDM runs for sets of two and three states whose concentration surfaces would be sufficiently far from one another such that they could be separated and attributed to their source-state. Our separation algorithm deliberately omitted a small fraction of total premature deaths to ensure sufficient separation of concentration surfaces and attribution to the appropriate source-state. This omission was less than 10% for each run, providing a modest downward bias in total premature deaths, but potentially greater biases for individual states included in multi-state runs. Similarly, we had to limit modeling to 2 months—January and July—chosen to be representative of opposing meteorological and atmospheric conditions. Choosing only 2 months requires us to assume that each of January and July reasonably represents half of the year, and that the average of these 2 months reasonably represents annual patterns. This approach has been used in previous studies and has been shown to represent seasonal and annual conditions appropriately, and our modeling of baseline concentrations showed only modest differences in comparison with full annual runs (< 5% on a domain average basis for both PM_2.5_ and O_3_, represented in Figure S5), but will have greater uncertainty than annual runs in predicted concentrations.

Outlier health damage function values appear in states with very low emissions. For example, Idaho emits 0.02 tons per year of primary PM_2.5_ from EGUs, far less than other states. These small emissions lead to very low modeled health impacts (0.05 deaths) over the course of a year, so the influence on total premature mortalities across the United States is miniscule, but the premature mortalities per ton emitted are much higher than anticipated. There may be an issue with utilizing CMAQ-DDM in discerning sensitivity of ambient concentrations to these miniscule emissions values, which is only pointed out in assessing the health damage function as normalized by these small emissions. This indicates there may be a lower limit on emissions when applying CMAQ-DDM in this manner.

Calculation of total premature mortalities and health damage functions relies upon accurate population and baseline mortality values, which were obtained as county-wide values and spatially joined to CMAQ’s 36 km × 36 km grid cells assuming uniform population characteristics. As population density is not uniform across a county, this assumption may have led to misattributed premature mortalities and health damage functions in specific grid cells. Because of the large spatial domains over which health impacts occur, these uncertainties are likely modest, although sources in dense urban areas with relatively small downwind populations could exhibit greater errors, especially for primary pollutants where the spatial domain of impact is smaller. Concentration-response functions contain uncertainty not presented within our analysis, but all values would scale linearly and conclusions about variability would be unaffected.

A considerable strength of our modeling platform is that precursor-specific findings along with characterization of background concentrations could allow for sensitivity analyses on these assumptions in future analyses. Although our analysis includes a number of uncertainties including those from use of the National Emissions Inventory, meteorological fields used, and CMAQ atmospheric model, we have not constructed distributions around our output values or formally propagated uncertainty. This is in part because of the complexity in quantifying CMAQ-DDM uncertainty for individual sources, and because of our focus on relative comparisons within this manuscript, but remains a limitation in interpreting and applying our results.

## Conclusions

In this study, we generated a novel set of estimates of both health impacts and health damage functions for RC and EGUs for the continental United States. We attribute premature deaths to emissions by source-state and precursor pollutant, which has not been done previously. These estimates can be used to address strategic emissions control policies on a state-by-state basis. Health damage functions can be used to determine which targeted emissions reductions will have the largest health benefits, an important part of creating efficient control strategies and designing SIPs that optimize health. Our use of CMAQ-DDM coupled with a complex image segmentation technique to isolate impacts of individual states can be extended to other source sectors, and source-based health damage functions can allow for understanding of how emissions impact health in a manner that can be helpful for state and federal policy makers.

## Supplemental Material

(2.2 MB) PDF
