# Supplementary material for: Estimating State-Specific Contributions to PM2.5- and O3-Related Health Burden from Residential Combustion and Electricity Generating Unit Emissions in the United States
Source: Environ Health Perspect. 2016 Sep 2;125(3):324–32. doi: 10.1289/EHP550 (PMC5332198; doi:10.1289/EHP550)
Supplement: (2.2 MB) PDF [file EHP550.s001.acco.pdf]

Environ Health Perspect
DOI: 10.1289/EHP550

Note to readers with disabilities: *EHP* strives to ensure that all journal content is accessible to all readers. However, some figures and Supplemental Material published in *EHP* articles may not conform to 508 standards due to the complexity of the information being presented. If you need assistance accessing journal content, please contact ehp508@niehs.nih.gov. Our staff will work with you to assess and meet your accessibility needs within 3 working days.

Supplemental Material

Estimating State-Specific Contributions to $PM_{2.5}$- and O_3-Related Health Burden from Residential Combustion and Electricity Generating Unit Emissions in the United States

Stefani L. Penn, Saravanan Arunachalam, Matthew Woody, Wendy Heiger-Bernays, Yorghos Tripodis, and Jonathan I. Levy

Table of Contents

Table S1. Grouping of sensitivity parameters for $PM_{2.5}$ and O_3

Figure S1: EGU emissions in the continental US by Tier 2 description (year 2005)

Table S2: EGU emissions by state in 2005 (tons/year), sorted by SO_2 emissions

Figure S2: RC emissions by fuel type across the continental US (year 2005)

Table S3. RC emissions by state in 2005 (tons/year), sorted by primary $PM_{2.5}$ emissions

Table S4. CMAQ-DDM run groups for RC and EGUs. States with #1 and #2 were subdivided to reflect different electricity dispatch regions

Table S5. RC-related deaths per year by state for each precursor-pollutant pair (n = 49)

Table S6. EGU-related deaths per year by state for each precursor-pollutant pair (n = 49

Figure S3. RC-related emissions of primary $PM_{2.5}$ by grid cell in January (left panel) and July (right panel)

Figure S4. EGU-related emissions of SO_2 by grid cell in January (left panel) and July (right panel)

Table S7. RC health damage functions (mortality risk per 1,000 tons of emissions) by state in January. States with the 5 lowest emissions for each pollutant are noted in italics. These values should be interpreted cautiously

Table S8. RC health damage functions (mortality risk per 1,000 tons of emissions) by state in July. States with the 5 lowest emissions for each pollutant are noted in italics. These values should be interpreted cautiously

Table S9. EGU health damage functions (mortality risk per 1,000 tons of emissions) by state in January. States with the 5 lowest emissions for each pollutant are noted in italics. These values should be interpreted cautiously

Table S10. EGU health damage functions (mortality risk per 1,000 tons of emissions) by state in July. States with the 5 lowest emissions for each pollutant are noted in italics. These values should be interpreted cautiously

Figure S5. Scatterplots showing the relationship between health damage functions for RC (x-axis) and EGUs (y-axis) for individual source states related to primary $PM_{2.5}$ (panel a), NOx related to $PM_{2.5}$(panel b), SO_2 related to $PM_{2.5}$ (panel c), VOC related to $PM_{2.5}$ (panel d), and O_3 (panele) for both January (left panel) and July (right panel.

Figure S6. Domain-average O_3 and $PM_{2.5}$, compared for different periods of CMAQ simulations. (Qtr1 represents Jan-Mar, Qtr2 represents Apr-Jun, Qtr3 represents Jul-Sep and Qtr4 represents Oct-Dec)

CMAQ Modeling

We used the WRF-SMOKE-CMAQ modeling system as described below to link emissions precursors (Table S1) by source sector and source-state with $PM_{2.5}$ and O_3 concentrations. This modeling platform is the same as that used in Levy et al (2016), and Boone et al (2016), and we provide a brief summary below.

Meteorological Inputs:

We created meteorological inputs from the Weather Research Forecast (WRF) model. For this study, WRF version 3.6.1 (Skamarock et al. 2008) was used to downscale NASA's Modern-Era Retrospective Reanalysis (hereafter, MERRA) to produce high-fidelity weather for CMAQ for the year 2005. MERRA is a global reanalysis with a horizontal grid resolution of 0.5° deg. x 0.67° deg. with 72 vertical levels extending to 0.01hPa. MERRA assimilates many observations, including NASA satellite products, into the reanalysis and is intended to improve the representation of the hydrologic cycle (Rienecker et al. 2011). We used a one-year spinup of WRF during 2004 using MERRA atmospheric forcing to bring the soil and atmospheric conditions, along with the atmosphere and lake surface temperatures, to come to equilibrium. A one-way nested approach is used to downscale MERRA to 36-km over the Contiguous United States (CONUS). WRF was run with a 35-layer configuration that extended up to 50 hPa. We evaluated WRF using observed datasets, and the model performance was in the general ranges of other regional-scale applications, and described in Boone et al (2016).

Emissions Inputs:

We processed emissions inventories from the US EPA's National Emissions Inventories (NEI) version 4.3 modeling platform for the year 2005 (US EPA, 2011) through the Sparse Matrix Operator Kernel Emissions (SMOKE) modeling system (Houyoux et al, 2000). The state-wide emissions summaries for each of EGUs and RC source sectors for the key precursor pollutants are provided in Tables S2 and S3. Figure S1 shows nation-wide EGU emissions broken down by fuel type, and Figure S2 shows nation-wide RC emissions broken down by fuel type, as reported in the NEI.

Table S1. Grouping of sensitivity parameters for $PM_{2.5}$ and O_3.

Group	Chemical Species	Species Name
PSO4	PSO4	Primary Sulfate
POC	POC	Primary Organic Carbon
PEC	PEC	Primary Elemental Carbon
VOC	ALD2	Acetaldehyde
	ALDX	Other Aldehydes
	ETH	Ethene
	ETHA	Ethane
	ETOH	Ethanol
	FORM	Formaldehyde
	IOLE	Internal Olefin Bond
	MEOH	Methanol
	OLE	Terminal Olefin Bond
	TOL	Toluene-Like

	XYL	Xylene-Like
SO2	SO2	Sulfur Dioxide
NOX	NO	Nitric Oxide
	NO2	Nitrogen Dioxide
	HONO	Nitrous Acid

Figure S1: EGU emissions in the continental US by Tier 2 description (year 2005).

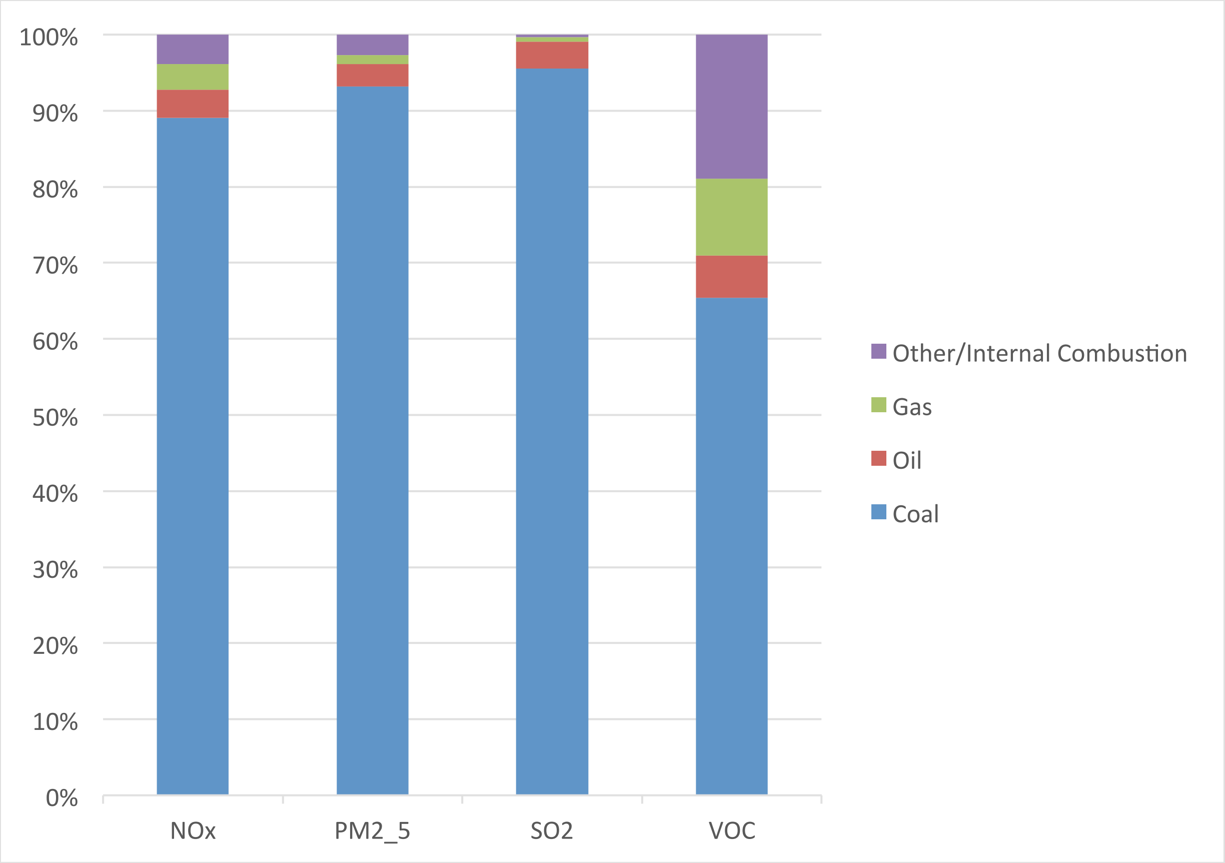

Table S2: EGU emissions by state in 2005 (tons/year), sorted by SO_2 emissions.

State	NOx	VOC	SO_2	$PM_{2.5}$
Ohio	258,940	1,766	1,116,100	53,572
Pennsylvania	176,890	1,154	1,002,200	55,547
Indiana	213,590	2,523	878,980	34,439
Georgia	111,280	1,325	616,060	28,057
Texas	176,170	3,851	534,950	21,464
North Carolina	111,580	936	512,230	16,967
Kentucky	164,780	1,482	502,730	19,830
West Virginia	159,950	1,141	469,460	26,377
Alabama	133,050	1,366	460,120	23,366

Florida	217,280	2,056	417,320	24,217
Michigan	120,030	1,232	349,880	11,022
Illinois	127,940	1,580	330,380	16,585
Missouri	127,430	1,597	284,380	6,472
Maryland	62,574	483	283,200	15,417
Tennessee	102,930	798	266,150	12,856
Virginia	62,793	656	220,290	12,357
South Carolina	52,657	533	218,780	14,455
New York	63,315	801	180,850	9,648
Wisconsin	72,170	980	180,200	5,233
North Dakota	76,381	763	137,370	6,398
Kansas	90,220	948	136,520	5,549
Iowa	72,806	536	130,260	8,898
Oklahoma	86,204	1,029	110,080	1,411
Louisiana	64,987	1,073	109,870	5,599
Minnesota	84,304	655	101,680	3,262
Wyoming	89,315	848	89,874	8,068
Massachusetts	25,134	584	84,234	3,110
Mississippi	45,166	574	75,047	2,029
Nebraska	52,426	676	74,955	1,246
Arkansas	35,407	480	66,384	1,688
Colorado	73,909	914	64,174	4,342
New Jersey	30,142	1,194	57,044	4,625
Nevada	47,297	524	53,363	3,341
Arizona	79,776	577	52,733	7,418
New Hampshire	8,827	136	51,445	2,586
Utah	65,261	368	34,813	5,055
Delaware	11,917	99	32,378	2,169
New Mexico	75,483	576	30,628	5,583
Montana	39,858	396	19,715	2,398
Oregon	9,383	141	12,304	412
South Dakota	15,650	106	12,215	390
Connecticut	6,865	307	10,356	562
Maine	1,100	60	3,887	52
Washington	17,634	248	3,409	2,396
District of Columbia	492	3	1,082	17
California	6,925	822	601	347
Rhode Island	545	35	176	10
Vermont	297	22	9	37
Tribal Data	78	133	3	0
Idaho	19	0	0	0

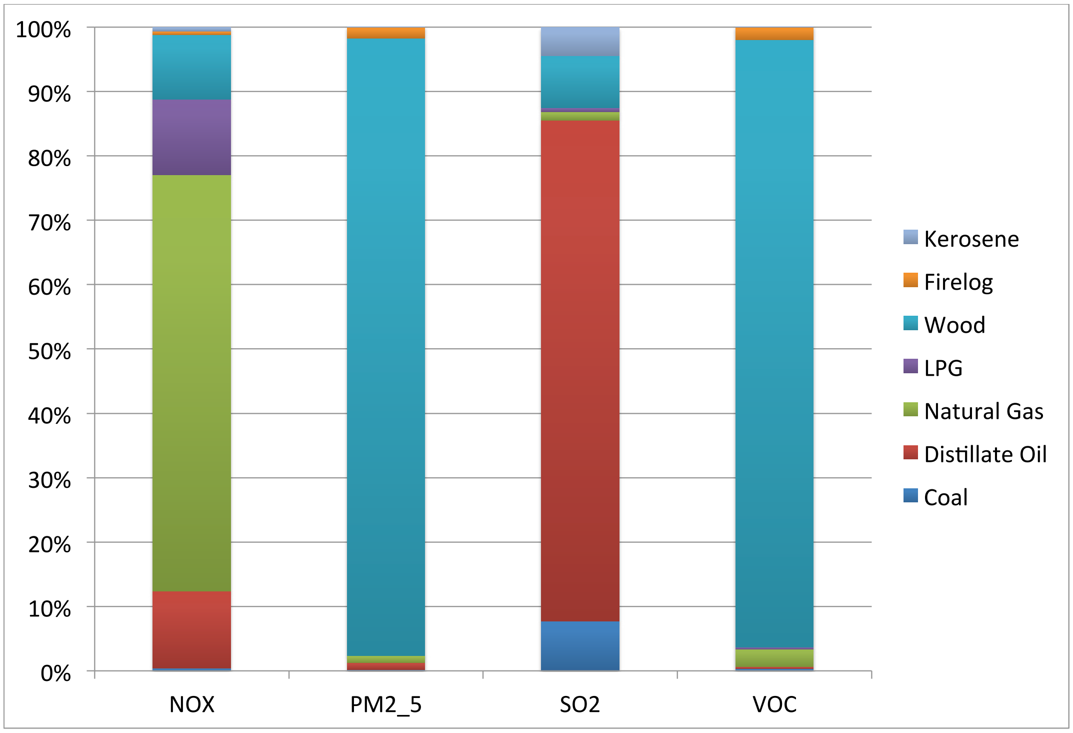

Figure S2: RC emissions by fuel type across the continental US (year 2005).

Table S3. RC emissions by state in 2005 (tons/year), sorted by primary $PM_{2.5}$ emissions.

State	NOx	VOC	SO_2	$PM_{2.5}$
California	27,234	20,416	1,480	40,100
Oregon	5,703	48,861	1,780	38,871
Wisconsin	11,176	25,590	3,491	20,995
Washington	7,087	27,289	1,460	19,900
New York	37,250	15,937	35,026	14,183
Massachusetts	15,749	25,231	20,196	14,096
Pennsylvania	22,495	19,628	30,333	13,115
Maine	4,612	20,128	7,571	12,941
Missouri	8,493	19,321	1,443	11,727
Minnesota	8,947	17,008	2,266	11,446
Colorado	6,925	14,988	370	11,440
North Carolina	7,082	17,182	4,939	10,749
Virginia	7,892	16,261	6,343	10,227
New Jersey	15,517	13,842	6,883	9,722
Ohio	20,590	14,253	1,625	9,283
Connecticut	8,016	15,154	12,354	9,144
Texas	11,262	15,412	988	8,654
Maryland	6,631	15,807	4,508	8,435

Michigan	18,855	15,007	2,131	8,322
New Hampshire	3,550	13,117	4,614	8,247
Illinois	24,481	13,987	906	7,702
Kentucky	4,597	12,365	1,490	7,605
Tennessee	4,748	11,583	946	7,121
Georgia	7,302	10,739	381	6,541
Iowa	5,229	9,812	2,641	5,977
South Carolina	2,858	12,339	1,388	5,499
Mississippi	3,615	12,317	500	4,970
Florida	2,508	6,974	543	4,563
Kansas	4,206	6,496	212	4,485
Indiana	9,076	7,729	1,928	4,430
Alabama	16,756	7,458	258	4,069
Vermont	2,029	5,654	1,553	3,799
Oklahoma	3,881	5,254	118	3,155
West Virginia	2,226	5,278	1,259	3,128
Montana	1,457	4,870	241	3,040
Arkansas	3,032	3,739	52	2,498
South Dakota	1,224	3,805	351	2,381
Idaho	1,760	3,349	785	2,370
Louisiana	2,943	3,857	73	2,333
Nebraska	2,555	3,542	145	2,139
Arizona	2,054	3,310	44	2,075
North Dakota	1,277	3,311	758	2,065
Utah	4,492	2,480	828	1,995
New Mexico	2,181	2,639	58	1,577
Wyoming	871	2,363	257	1,473
Nevada	1,871	1,505	152	1,098
Delaware	1,225	2,401	945	799
Rhode Island	2,157	678	2,847	521
District of Columbia	820	180	347	100

Community Multiscale Air Quality (CMAQ) model

We used the Community Multiscale Air Quality (CMAQ) model version 4.7 (Byun and Ching, 1999; Byun and Schere, 2006) instrumented with the Decoupled Direct Method in Three Dimensions (DDM-3D) (Dunker et al, 1984; Napelenok et al, 2006). The model was configured with the Carbon Bond chemical mechanism for gas-phase species (CB-05) with aerosol treatment version 5 (aero5). Detailed description of the scientific advances in CMAQ v4.7 and the corresponding evaluation are described in Foley et al (2010). For this study, we ran CMAQ for the January and July months with an 11 day spinup for each month. The January and July months are representative of winter and summer months respectively. The initial and boundary conditions for CMAQ were generated from a global simulation for the year 2005 using the CAMChem model (Lamarque et al, 2011).

This approach of using representative months to represent an entire year has been used in several previous studies including by the US EPA as well as by Ashok et al (2011) and Foley et al (2014), where a Response Surface Model (RSM) was developed using hundreds of CMAQ simulations. In Figure S6, we provide a comparison of domain-average CMAQ predictions for a typical base case simulation over the continental US using a 2-month average (from January and July) compared against a true 12-month average for O_3 and $PM_{2.5}$. In addition, the 4 quarterly averages through the year are also included to show the seasonal variations. From this analysis, we see that the 2-month average approximates a true 12-month average within 5% for both pollutants studied.

In addition, we evaluated the CMAQ outputs against four routine US air quality networks – for O_3, the Air Quality System (AQS) network; and for $PM_{2.5}$ measurements, the Clean Air Status and Trends Network (CASTNet), Interagency Monitoring of Protected Visual Environments (IMPROVE) network, and the SouthEastern Aerosol Research and Characterization (SEARCH) Network. The summary of this evaluation (Boone et al, 2016) showed that CMAQ performance is within the broad bounds of regional-scale model applications as summarized by Simon et al (2012).

For computing DDM-based sensitivities, we grouped the emissions precursors as shown in Table S1. While $PM_{2.5}$ had 6 groups of precursors, O_3 had only two, i.e., NO_x and VOC. For each DDM group listed in Table S4, we created individual emissions input files that had either EGU or RC emissions from the states within that group for these 6 groups of precursors.

We then post-processed the outputs to compute the first order sensitivities of $PM_{2.5}$ and O_3 to each individual precursor group for each of the two source sectors (EGU and RC), which served as inputs to the image separation algorithm described below.

Table S4. CMAQ-DDM run groups for RC and EGUs. States with #1 and #2 were subdivided to reflect different electricity dispatch regions.

EGU Group	RC Group
CO	RI, CO, TN
ME, MO#1	ME, MO, ID
NH, WI#1	NH, WI
FL, NE	NJ, FL, NE
SC, SD#1	SC, SD
CT, KS, WA	CT, KS, WA
MA, WY#1	MA, AL, WY
OK	VT, OK
DE, ND	ND
DC, OR	DC, OR
PA, TX#1	PA, TX
MN, NY	DE
LA#1, VA#1, MT	LA, VA, MT
GA	GA
TX#2, WV	NM, WV
IA	IA, MD
IL#2, UT	IL, UT
IN, AZ	IN, AZ
OH, CA	OH, CA
AR#1, NV#1	NC, MN
WI#2	AR, NV
IL#2	KY
AR#2, NV#2	NY
VA#2, SD#2	MI
NJ, WY#2	MS
TN	
AL	
KY	
MS	
MI	
MD	
NC	
MO#2	
LA#2	
RI	
ID	
VT	

NM#1	
NM#2	

Image Segmentation Algorithm

DDM output is visualized as 112 row x 148 column 36km x 36km grid cells overlaid on the continental US. Based on this concentration-response surface format, image segmentation techniques were used to separate individual emissions plumes from one another within a group's DDM output surface using MATLAB 8.1.0, R2013a (MathWorks, Natick, MA). For each emitted precursor / ambient pollutant relationship for each group and month, a region growing algorithm was developed to determine the emissions regions attributable to each state or EGU region. The following steps were followed:

1. Find maximum sensitivity "near" the centroid of the state, where "near" = within a radius of 288 km. These locations were used as the seed locations for each region. For pollutants with negative sensitivities (i.e., the relationship between NOx emissions and ambient O_3 concentrations), the minimum concentration (i.e., most negative) near each state was also found.

2. Use maximum concentration as the first positive threshold value for region growing. For pollutants with negative sensitivities, the initial negative threshold was set as the minimum concentration in that group run.

The region growing algorithm was run iteratively. The absolute value of the threshold value(s) were decreased (brought closer to zero) by 10% of each iteration until:

1. The number of mortalities captured by the sum of the emissions regions is greater than a chosen threshold (95% for RC, 90% for EGUs) of the total number of mortalities as predicted by the full group of states,

AND

2. The threshold value is less than a specific percent (25%) of the maximum nearby concentration for all of the states.

Once the regions captured >95%/90% of the total group-wise predicted mortalities, only states whose maximum nearby concentration was >25% of the threshold value were allowed to continue to grow.

The region growing algorithm is as follows:

For each state (in increasing order of maximum nearby concentration) {

Add cell at state centroid location to "queue".

While "queue" is not empty {

Search 8 nearest neighbors of grid cell at top of queue, add any cells that are:

Concentration is Greater than positive threshold value *OR* less than negative threshold values (for the relationships between VOC and O_3, VOC and $PM_{2.5}$, and NOx and O_3).

AND

Not in (or within 1 cell of the boundary of) another state's region from the previous iteration.

Remove current cell from queue.

}

}

In post-processing, regions were masked by the land of the contiguous United States and holes within each region were filled to form contiguous emissions areas for each individual state.

Table S5. RC-related deaths per year by state for each precursor-pollutant pair (n = 49).

All values are rounded to two significant figures. Sums may not add due to rounding.

State	PEC – $PM_{2.5}$	POC– $PM_{2.5}$	PSO_4– $PM_{2.5}$	NOx– $PM_{2.5}$	SO_2– $PM_{2.5}$	VOC– $PM_{2.5}$	O_3 - NOx	O_3 - VOC	Total by State
AL	6.1	47	1.4	12	1.2	2	-31	15	55
AR	3	26	0.43	3.4	0.34	1.1	0.91	17	52
AZ	0.72	6.8	0.027	0.88	0.008	0.017	1.2	1.4	11
CA	110	620	58	96	56	56	-49	33	980
CO	2.8	27	0.096	1.6	0.0056	0.024	-2.4	3.5	32
CT	85	340	63	55	53	58	-34	28	650
DC	0.11	0.3	0.14	0.16	0.075	0.15	-0.72	1.7	1.9
DE	35	59	32	32	31	32	-2.1	5.6	230
FL	4.3	34	1.2	3.7	0.87	0.74	3.3	9.7	58
GA	9.1	85	0.51	4.1	0.14	1.6	-9.3	28	120
IA	2.8	37	0.63	16	0.17	1.9	-11	25	72
ID	0.18	1.7	0.024	0.63	0.0033	-0.041	1.1	0.073	3.7
IL	14	120	0.65	17	0.33	1.8	-64	48	140
IN	9.5	82	0.85	8.7	0.3	2.8	-14	25	120
KS	2.3	30	0.0074	4.4	1.5	0.56	-8.4	21	51
KY	9.1	82	0.45	2.3	0.43	2.2	-9.3	31	120
LA	2.2	24	0.12	1.2	0.06	0.28	-3.3	9.7	34
MA	49	200	36	38	32	35	-23	26	390
MD	120	460	84	83	79	86	-29	48	930

**ME**	11	48	6.7	9.7	6.2	6.9	2	5.9	96
**MI**	9.5	88	0.52	4.2	0.14	1.7	-74	57	87
**MN**	11	73	4.2	29	3.2	7	-21	44	150
**MO**	9.7	91	0.38	7.4	0.07	3.1	-8.6	55	160
**MS**	3	33	0.48	1.6	0.31	0.52	0.19	27	66
**MT**	0.16	1.6	0.013	1.7	0.018	-0.087	-0.03	0.15	3.5
**NC**	15	140	1.5	5.7	1.5	2.9	-5.3	38	200
**ND**	1.1	9.3	0.26	5.8	0.15	0.57	-1.5	7.6	23
**NE**	0.53	5.2	0.044	6.4	0.0067	0.53	-0.63	4.9	17
**NH**	26	110	17	22	16	19	-2.6	35	240
**NJ**	59	540	11	-4.4	0.85	7.5	-89	29	550
**NM**	0.29	1.6	0.025	0.66	0.022	0.049	2.1	1.2	6
**NV**	0.42	3.5	0.035	1.7	0.029	0.018	0.55	0.83	7.1
**NY**	130	360	120	130	99	100	-48	37	910
**OH**	290	490	260	270	260	270	-41	48	1800
**OK**	3.4	32	0.11	3	0.034	1.3	-5.9	20	54
**OR**	24	230	1.6	10	0.18	-1.9	-3.5	16	280
**PA**	76	400	31	16	5.6	11	-130	49	460
**RI**	0.46	2.7	0.58	1.1	0.15	0.051	-1.8	0.41	3.7
**SC**	11	74	3	4.5	2.4	4	-2.7	37	130
**SD**	0.84	8.3	0.12	5.2	0.027	0.45	-0.97	4.9	19
**TN**	8.8	79	0.37	2.5	0.29	2	-5.6	36	120
**TX**	8.1	79	0.26	4	0.38	2.1	-17	35	110
**UT**	0.67	4.6	0.28	1.7	0.28	0.26	-0.8	0.66	7.6
**VA**	19	160	2.4	5.5	1.7	3.4	-13	34	210
**VT**	4.5	39	0.74	5	0.16	1.1	0.38	10	61
**WA**	9.2	87	0.33	3.7	0.031	-0.11	-7	4.3	97
**WI**	32	300	2	20	0.25	11	-35	95	430
**WV**	4.4	36	0.36	1.4	0.36	0.85	-5.1	8.7	47
**WY**	0.025	0.23	0.00016	0.083	0.0013	-0.022	-0.049	0.27	0.54
**Sum**	1,200	5,800	740	960	660	740	-800	1,100	10,000

Table S6. EGU-related deaths per year by state for each precursor-pollutant pair (n = 49). All values are rounded to two significant figures. Sums may not add due to rounding.

State	PEC – $PM_{2.5}$	POC– $PM_{2.5}$	PSO_4– $PM_{2.5}$	NOx– $PM_{2.5}$	SO_2– $PM_{2.5}$	VOC– $PM_{2.5}$	O_3 - NOx	O_3 - VOC	Total by State

**AL**	17	15	86	110	370	7.1	86	0.16	690
**AR**	3.3	3.1	15	34	81	2.7	49	0.071	190
**AZ**	0.45	0.48	1	9.9	53	0.26	12	0.005	77
**CA**	8.7	7.7	6.4	11	6.4	7	3.3	0.31	51
**CO**	1.2	1	4	14	19	0.79	21	0.059	60
**CT**	1.8	1.6	6.3	3.8	45	1.5	3	0.12	63
**DC**	0.53	0.45	0.64	1.1	3.3	0.34	0.36	0.0013	6.7
**DE**	4.6	4.8	24	24	56	2	7.3	0.077	120
**FL**	37	34	140	120	380	27	71	0.46	810
**GA**	15	13	110	81	370	6.3	510	0.89	1100
**IA**	9.1	7.7	40	150	130	4.7	16	0.11	350
**ID**	0.015	0.016	0.016	0.024	0.016	0.016	0.049	0.0011	0.15
**IL**	16	13	88	130	440	5.3	44	0.32	740
**IN**	56	50	240	330	1200	33	85	0.44	2000
**KS**	4.9	4.5	23	66	85	3.5	25	0.14	210
**KY**	32	29	140	200	580	21	83	0.23	1100
**LA**	11	12	27	36	87	7.9	29	0.19	210
**MA**	6.2	5.7	16	17	36	5.1	8.1	0.09	93
**MD**	26	21	110	95	290	12	32	0.092	590
**ME**	0.21	0.2	0.36	1.8	9.3	0.21	0.46	0.0098	13
**MI**	14	12	110	200	640	6	15	0.36	1000
**MN**	0.76	0.38	11	96	30	0.22	-2.1	0.11	140
**MO**	15	15	61	110	270	13	46	0.23	540
**MS**	3.6	2.9	10	22	54	2.2	27	0.11	120
**MT**	0.019	0.034	0.12	9.3	0.18	0.0018	-3.6	0.0023	6.1
**NC**	9.4	7.3	110	100	340	1.3	61	0.097	640
**ND**	3	12	16	180	44	2.7	-6	0.097	250
**NE**	0.52	0.53	4.6	48	43	0.58	7.4	0.035	100
**NH**	2.7	2.5	10	8.6	40	1.8	2.9	0.02	68
**NJ**	15	11	34	30	66	5.9	3.4	0.4	170
**NM**	0.51	0.38	0.55	11	8.3	0.059	110	0.15	130
**NV**	1.1	0.96	2.9	16	10	0.65	10	0.0096	42
**NY**	82	79	140	160	240	72	4.8	0.4	790
**OH**	40	31	290	350	1500	4.5	85	0.24	2300
**OK**	11	12	19	63	100	10	44	0.22	260
**OR**	0.45	0.44	1.1	7.2	2.3	0.41	2.7	0.0084	15
**PA**	61	50	330	290	1200	16	64	0.23	2000
**RI**	0.5	0.44	0.42	0.58	0.46	0.43	1.1	0.066	3.9
**SC**	8	6.6	50	38	110	2.8	33	0.071	250
**SD**	0.014	0.0097	1	35	0.42	0.22	-1	0.016	36
**TN**	12	10	61	82	230	6.9	64	0.11	460

**TX**	26	50	110	71	380	21	54	0.5	710
**UT**	1.7	1.5	2.2	26	3.9	1.1	8.9	0.007	45
**VA**	13	10	63	71	190	4.7	49	0.17	400
**VT**	0.027	0.13	0.034	0.44	0.017	0.02	1.1	0.041	1.8
**WA**	0.8	1.2	2.5	7.2	1.4	0.13	5.6	0.028	19
**WI**	13	12	56	130	220	9.1	150	1.7	600
**WV**	17	16	130	210	530	3.9	110	0.092	1000
**WY**	0.23	0.21	1.1	21	14	0.16	12	0.014	48
**Sum**	600	570	2,700	3,800	10,000	340	2,000	9.3	21,000

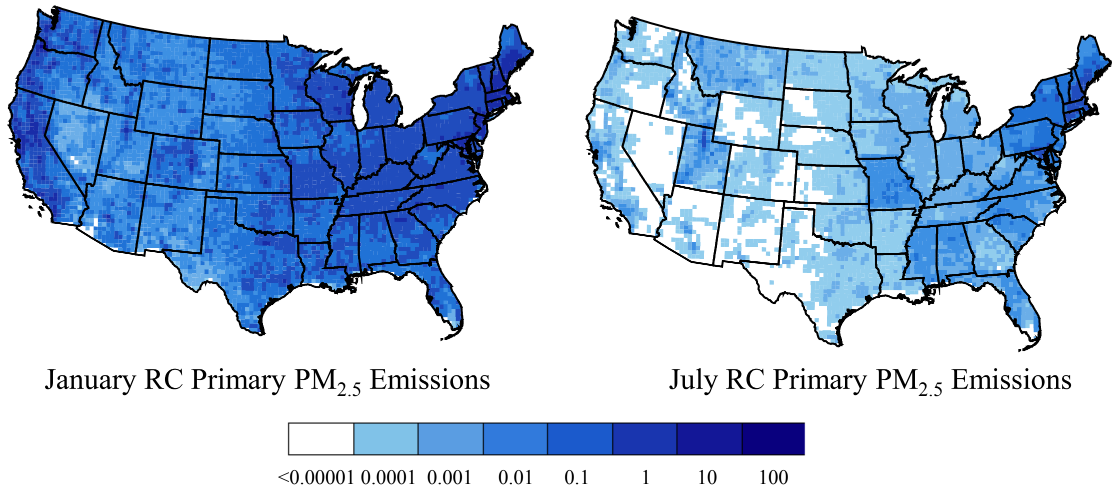

Figure S3. RC-related emissions of primary $PM_{2.5}$ by grid cell in January (left panel) and July (right panel).

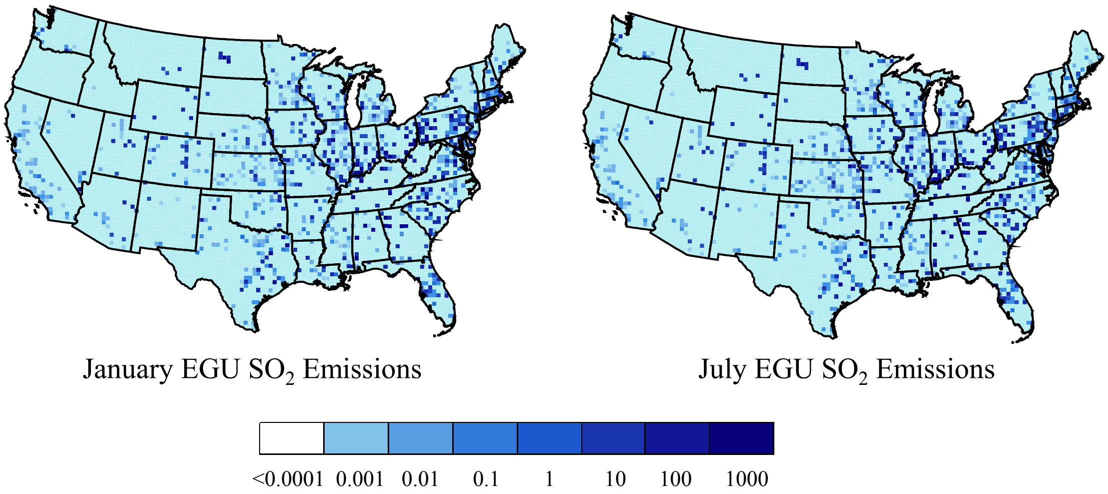

Figure S4. EGU-related emissions of SO_2 by grid cell in January (left panel) and July (right panel).

Table S7. RC health damage functions (mortality risk per 1,000 tons of emissions) by state in January. States with the 5 lowest emissions for each pollutant are noted in italics. These values should be interpreted cautiously.

	**PEC-$PM_{2.5}$ Jan**	**POC-$PM_{2.5}$ Jan**	**PSO_4-$PM_{2.5}$ Jan**	**NOx-$PM_{2.5}$ Jan**	**SO_2 - $PM_{2.5}$ Jan**	**VOC - $PM_{2.5}$ Jan**	**NOx – O_3 Jan**	**VOC - O_3 Jan**
**AL**	27.18	22.89	62.90	0.57	4.38	0.50	-2.41	3.92
**AR**	14.97	13.71	26.18	1.01	4.83	0.35	-1.96	5.15
**AZ**	5.30	5.33	2.35	0.40	0.06	0.01	0.32	0.57
**CA**	38.68	22.30	219.49	3.84	32.47	3.39	-2.83	1.97
**CO**	6.24	6.28	2.44	0.22	0.01	0.00	-0.48	0.52
**CT**	129	57.71	353	6.01	3.64	5.89	-4.15	2.07
**DC**	*13.94*	*5.75*	*41.73*	*0.09*	*0.08*	*1.69*	*-1.03*	*20.22*
**DE**	*720*	*148*	*2570*	*26.29*	*30.73*	*26.87*	*-5.11*	*2.60*
**FL**	17.34	15.29	23.19	1.81	1.36	0.18	-0.48	2.38
**GA**	20.63	20.65	13.35	0.42	0.19	0.21	-2.05	3.46
**IA**	10.98	16.82	23.12	3.00	0.03	0.43	-2.95	5.64
**ID**	1.81	1.86	1.89	0.60	0.00	-0.03	-0.11	0.05
**IL**	30.60	30.30	12.06	0.54	0.13	0.22	-2.76	5.82
**IN**	35.21	35.12	21.38	0.62	0.07	0.61	-2.32	5.46
**KS**	9.24	12.66	0.30	0.97	6.72	0.13	-2.27	5.22
**KY**	23.17	23.49	11.61	0.29	0.09	0.33	-3.11	4.58
**LA**	11.42	13.22	7.19	0.37	0.22	0.08	-1.66	2.75
**MA**	49.57	21.22	233	2.09	1.35	2.15	-1.54	1.21
**MD**	196	79.10	957	10.83	15.42	8.62	-4.86	3.39
**ME**	11.82	5.91	53.06	1.99	0.75	0.48	-0.43	0.37

**MI**	17.43	17.69	7.21	0.16	0.03	0.08	-3.59	5.58
**MN**	26.08	18.04	72.90	3.15	1.26	1.00	-2.61	6.28
**MO**	16.95	17.41	5.67	0.74	0.00	0.30	-1.26	5.38
**MS**	5.83	7.12	5.05	0.19	0.28	0.04	-0.82	2.01
**MT**	1.14	1.17	0.94	1.16	0.00	-0.04	-0.06	0.06
**NC**	21.62	23.23	18.51	0.59	0.12	0.25	-2.73	3.36
**ND**	12.57	12.69	19.53	4.85	0.15	0.47	-1.80	5.35
**NE**	*5.87*	*6.11*	*4.85*	*2.60*	*0.03*	*0.32*	*-0.72*	*3.01*
**NH**	45.65	20.69	199	5.95	3.07	2.05	-1.60	3.81
**NJ**	83.78	84.27	87.39	-0.32	0.05	0.72	-2.74	2.73
**NM**	3.97	2.43	1.91	0.28	0.16	0.03	0.64	0.96
**NV**	7.46	6.78	3.38	0.83	0.09	0.01	0.08	1.01
**NY**	129.35	42.24	328	3.83	2.45	14.50	-2.74	4.53
**OH**	405	79.46	2900	12.27	158	24.52	-3	4.43
**OK**	14.54	14.60	5.16	0.69	0.07	0.31	-2.04	4.67
**OR**	12.56	12.63	10.54	1.98	0.05	-0.06	-0.87	0.51
**PA**	61.09	59.70	97.53	0.39	0.07	0.90	-5.98	4.08
**RI**	*11.56*	*11.47*	*16.82*	*0.44*	*0.03*	*0.14*	*-1.09*	*1.13*
**SC**	31.56	26.18	48.02	1.41	1.70	0.49	-2.8	4.53
**SD**	9.28	9.90	11.21	4.68	0.06	0.28	-1.24	3.03
**TN**	20.96	20.94	10.32	0.37	0.09	0.27	-1.22	4.86
**TX**	11.95	12.38	4.04	0.31	0.25	0.17	-1.64	2.74
**UT**	5.74	3.71	16.32	0.34	0.29	0.15	-0.25	0.41
**VA**	33.31	33.14	25.00	0.41	0.08	0.37	-1.65	3.59
**VT**	17.30	17.34	16.54	2.47	0.04	0.27	-1.31	2.48
**WA**	9.62	9.80	4.01	0.52	0.01	-0.01	-0.54	0.28
**WI**	25.55	25.91	15.16	1.65	0.03	0.61	-3.57	5.41
**WV**	25.70	26.53	17.87	0.41	0.06	0.30	-3.01	3.13
**WY**	*0.40*	*0.43*	*0.02*	*0.09*	*0.00*	*-0.02*	*-0.10*	*0.26*

Table S8. RC health damage functions (mortality risk per 1,000 tons of emissions) by state in July. States with the 5 lowest emissions for each pollutant are noted in italics. These values should be interpreted cautiously.

	PEC-$PM_{2.5}$ Jul	POC-$PM_{2.5}$ Jul	PSO_4-$PM_{2.5}$ Jul	NOx - $PM_{2.5}$ Jul	SO_2 - $PM_{2.5}$ Jul	VOC-$PM_{2.5}$ Jul	NOx - O_3 Jul	VOC - O_3 Jul
**AL**	21.14	18.77	54.33	1.47	1.40	0.30	14.87	0.16
**AR**	56.39	25.34	72.66	0.87	6.59	51.06	14.24	3.25
**AZ**	4.86	2.20	3.88	0.00	2.85	0.91	2.76	0.11
**CA**	6.06	18.27	18.21	0.25	1.08	0.04	3.43	3.92
**CO**	2.68	3.14	13.86	0.03	0.07	3.06	2.35	0.04

**CT**	65.46	47.25	239	2.93	3.28	0.48	6.27	2.86
**DC**	*125*	*62.00*	*275*	*2.76*	*3.75*	*24.89*	*9.75*	*6.13*
**DE**	*108*	*70.92*	*375*	*7.82*	*5.52*	*1.14*	*20.97*	*3.63*
**FL**	10.56	10.32	17.00	0.36	2.52	0.18	5.97	1.15
**GA**	18.59	21.03	55.20	1.23	0.81	0.02	11.05	1.97
**IA**	1.27	1.19	24.78	1.26	1.00	10.68	6.79	0.01
**ID**	0.75	0.98	1.98	0.01	0.03	1.61	1.77	0.00
**IL**	32.21	46.52	52.24	1.76	2.54	0.09	6.89	5.93
**IN**	39.72	31.67	87.36	3.52	2.64	2.92	10.95	1.09
**KS**	4.28	4.08	7.24	0.31	3.39	120	5.12	0.02
**KY**	22.59	24.68	92.25	2.34	1.72	5.62	13.50	0.36
**LA**	4.36	4.45	22.49	0.22	3.05	6.07	7.56	0.20
**MA**	21.92	16.15	99.72	0.95	1.27	0.17	3.68	1.41
**MD**	82.22	59.06	384	7.89	7.41	0.22	14.01	4.13
**ME**	5.30	4.40	21.92	0.28	0.44	0.09	7.13	0.33
**MI**	20.39	17.56	24.41	1.35	0.58	0.02	6.57	8.03
**MN**	20.95	7.80	33.96	0.86	0.88	22.44	4.63	1.49
**MO**	13.15	12.83	18.94	1.16	0.96	0.07	5.64	0.06
**MS**	4.36	5.43	6.33	0.21	0.22	0.09	7.05	0.09
**MT**	0.51	0.96	1.01	0.00	2.44	0.03	0.53	0.00
**NC**	18.45	19.26	56.38	1.59	0.98	5.05	12.25	0.54
**ND**	30.94	14.12	51.77	0.35	1.13	0.08	3.38	0.92
**NE**	1.92	2.42	5.37	0.25	0.27	30.39	2.85	0.00
**NH**	12.85	10.03	50.84	0.91	0.96	0.14	7.46	0.71
**NJ**	71.41	73.14	162	1.46	1.58	0.12	3.84	4.88
**NM**	71.21	5.74	97.12	0.12	3.18	47.10	3.29	2.79
**NV**	*69.42*	*3.10*	*135*	*0.17*	*1.88*	*160*	*3.47*	*10.46*
**NY**	26.83	19.43	93.85	0.98	1.62	0.17	3.46	1.11
**OH**	211	285	1220	4.49	9.09	265	10.93	0.24
**OK**	13.45	4.51	58.13	0.39	0.97	32.94	7.95	0.13
**OR**	9.13	10.10	28.02	0.57	0.79	3.63	6.15	1.06
**PA**	53.09	38.99	145	4.08	1.94	0.08	12.47	1.75
**RI**	*16.29*	*15.83*	*45.86*	*0.42*	*0.46*	*0.14*	*8.83*	*1.62*
**SC**	21.62	20.10	38.62	1.19	0.92	0.17	14.96	0.32
**SD**	1.57	0.88	27.50	0.15	0.38	22.24	2.49	10.83
**TN**	17.95	18.96	57.30	1.50	1.21	5.53	6.74	0.10
**TX**	1.96	1.09	8.33	0.15	2.06	2.07	4.24	0.04
**UT**	2.97	3.04	4.49	0.01	0.13	0.13	2.08	0.15
**VA**	27.07	30.12	79.41	2.42	1.38	1.12	7.27	2.09
**VT**	9.03	8.56	16.35	0.44	0.50	0.09	9.84	0.43
**WA**	4.68	5.01	10.51	0.26	0.38	0.61	0.74	1.00

**WI**	5.50	0.30	26.69	1.19	1.23	0.10	5.82	4.13
**WV**	29.85	27.36	136	2.89	1.60	5.45	14.91	0.32
**WY**	*0.02*	*0.02*	*0.09*	*0.00*	*0.15*	*0.01*	*0.59*	*0.00*

Table S9. EGU health damage functions (mortality risk per 1,000 tons of emissions) by state in January. States with the 5 lowest emissions for each pollutant are noted in italics. These values should be interpreted cautiously.

	**PEC-$PM_{2.5}$ Jan**	**POC-$PM_{2.5}$ Jan**	**PSO_4-$PM_{2.5}$ Jan**	**NOx-$PM_{2.5}$ Jan**	**SO_2 - $PM_{2.5}$ Jan**	**VOC - $PM_{2.5}$ Jan**	**NOx - O_3 Jan**	**VOC - O_3 Jan**
**AL**	23.80	28.25	28.37	0.35	0.30	14.76	-0.59	0.41
**AR**	148	204	134	2.45	0.69	41.39	-0.20	1.42
**AZ**	2.90	3.50	1.68	0.27	0.04	1.65	0.05	0.05
**CA**	129	184	430	3.72	*22.78*	31.15	-0.46	0.84
**CO**	9.92	13.18	8.17	0.31	0.06	2.04	0.04	0.14
**CT**	117	161	39.51	0.74	0.32	10.22	-0.99	0.58
**DC**	*153*	*181*	*336*	*1.22*	*0.94*	*371*	*-0.53*	*1.41*
**DE**	66.81	107	64.83	0.66	0.28	58.50	-1.64	1.95
**FL**	69.48	91.22	29.46	0.81	0.66	35.44	-0.25	0.50
**GA**	19.58	23.13	37.67	0.45	0.26	15.14	-0.99	2.48
**IA**	46.31	57.84	58.35	3.58	0.22	34.22	-0.82	0.77
**ID**	*1181*	*1829*	*5234*	*1.60*	*45.15*	*40.02*	*0.93*	*1.95*
**IL**	83.76	97.35	63.80	0.75	0.27	37.23	-1.40	1.59
**IN**	64.33	80.71	39.46	0.80	0.17	48.20	-0.83	0.58
**KS**	59.95	75.35	36.31	1.07	0.15	15.61	-0.65	0.59
**KY**	63.71	82.14	44.28	0.55	0.19	45.65	-0.81	0.52
**LA**	101.17	78.51	87.10	1.77	0.93	41.41	-0.47	1.12
**MA**	66.63	95.77	25.85	0.76	0.12	18.44	-0.34	0.23
**MD**	56.67	68.10	41.92	0.73	0.14	70.99	-0.85	0.46
**ME**	*188*	*181*	*21.85*	*3.08*	0.31	*6.77*	*0.00*	*0.37*
**MI**	45.43	54.30	54.42	1.54	0.14	15.41	-0.86	0.73
**MN**	9.75	5.85	60.19	2.27	0.08	1.22	-0.62	0.61
**MO**	269	360	173	2.05	0.41	68.54	-1.55	1.32
**MS**	33.05	54.24	54.99	0.52	0.34	13.38	-0.38	0.61
**MT**	0.48	0.89	0.60	1.12	0.00	0.02	-0.78	0.04
**NC**	17.59	18.62	45.08	0.49	0.15	4.37	-0.59	0.38
**ND**	64.97	12.13	54.36	4.19	0.16	10.85	-0.39	0.39
**NE**	19.75	28.98	33.58	1.85	0.03	4.22	-0.26	0.26
**NH**	39.85	52.27	25.31	1.85	0.11	32.98	-0.63	0.30
**NJ**	81.36	95.04	54.45	0.52	0.24	13.51	-1.04	0.33
**NM**	3.75	5.48	14.15	0.29	0.67	1.02	0.36	1.53

**NV**	37.92	52.94	22.74	1.66	0.18	10.30	0.00	0.18
**NY**	371	513	127	3.88	0.81	208	-1.00	0.74
**OH**	21.69	23.03	20.55	0.41	0.07	7.46	-0.78	0.37
**OK**	246	200	158	1.17	0.37	44.83	-0.71	0.93
**OR**	49.51	66.06	35.95	1.58	0.13	8.52	-0.07	0.12
**PA**	36.98	42.52	35.44	0.78	0.09	38.13	-0.85	0.41
**RI**	*84.89*	*333*	*6100*	*1.64*	*4.51*	*18.99*	*-1.00*	*1.80*
**SC**	19.64	22.56	31.99	0.72	0.25	15.98	-0.51	0.45
**SD**	1.77	1.70	57.30	6.09	0.06	104	-0.68	0.59
**TN**	34.91	44.39	37.95	0.46	0.22	28.08	-0.70	0.53
**TX**	128	38.87	127	1.20	0.79	37.36	-0.91	0.92
**UT**	14.54	18.02	5.61	0.76	0.10	10.03	-0.15	0.07
**VA**	67.11	82.97	71.36	1.06	0.23	36.01	-1.46	1.41
**VT**	*18.70*	*14.57*	*12.72*	*2.79*	*0.69*	*0.61*	*-0.93*	*2.87*
**WA**	6.88	4.62	3.65	0.45	0.13	2.19	-0.09	0.01
**WI**	97.45	125	94.48	2.14	0.24	35.28	-2.06	2.98
**WV**	19.60	27.11	21.74	0.46	0.04	10.10	-0.55	0.28
**WY**	2.99	3.80	3.18	1.15	0.09	1.46	-0.14	0.14

Table S10. EGU health damage functions (mortality risk per 1,000 tons of emissions) by state in July. States with the 5 lowest emissions for each pollutant are noted in italics. These values should be interpreted cautiously.

	**PEC-$PM_{2.5}$ Jul**	**POC-$PM_{2.5}$ Jul**	**PSO_4-$PM_{2.5}$ Jul**	**NOx - $PM_{2.5}$ Jul**	**SO_2 - $PM_{2.5}$ Jul**	**VOC-$PM_{2.5}$ Jul**	**NOx - O_3 Jul**	**VOC - O_3 Jul**
**AL**	11.17	11.90	48.09	1.43	1.42	2.39	2.05	-0.02
**AR**	21.14	24.66	180	1.86	4.08	2.55	5.20	-0.04
**AZ**	0.32	0.41	1.17	0.00	2.07	0.01	0.28	-0.02
**CA**	14.27	13.46	5.49	0.27	*0.37*	1.04	1.27	0.44
**CO**	1.66	1.75	11.36	0.10	0.53	0.07	0.52	0.02
**CT**	48.32	58.53	69.59	0.39	9.59	2.87	2.04	0.54
**DC**	*52.06*	*51.82*	*228*	*3.39*	*5.57*	*72.05*	*2.19*	*0.32*
**DE**	31.82	41.79	130	3.55	3.40	7.53	2.89	0.57
**FL**	15.13	17.74	21.85	0.36	1.30	4.66	0.95	0.18
**GA**	7.12	7.53	46.23	1.09	1.02	2.05	10.59	-0.02
**IA**	9.13	9.30	40.33	1.08	1.72	1.07	1.09	0.03
**ID**	*3785*	*6018*	*17400*	*1.30*	*149*	*133*	*4.31*	*9.90*
**IL**	35.88	36.87	200	3.22	5.83	4.49	2.62	0.32
**IN**	17.22	18.60	108	2.45	2.77	4.03	1.62	0.10
**KS**	5.82	5.84	38.63	0.62	0.95	0.29	0.85	0.04
**KY**	16.89	18.72	101	2.04	2.27	4.82	1.84	0.04

**LA**	19.96	19.25	74.98	0.70	2.42	4.01	2.31	0.13
**MA**	25.41	33.87	41.42	0.65	0.81	5.76	1.03	-2.37E-03
**MD**	25.19	26.43	95.21	2.50	2.02	12.76	1.93	0.20
**ME**	*71.51*	*63.68*	*11.04*	*0.06*	5.30	*1.70*	*0.95*	*0.03*
**MI**	18.29	18.99	138	2.08	3.59	1.57	1.05	0.26
**MN**	2.00	1.43	20.18	0.50	0.50	0.04	0.40	0.02
**MO**	20.39	22.38	191	1.75	2.68	2.20	2.42	2.20E-03
**MS**	10.02	11.82	51.33	0.48	1.19	1.28	1.58	0.08
**MT**	0.13	0.16	0.55	0.00	0.01	0.00	0.10	4.79E-04
**NC**	10.21	10.36	91.18	1.52	1.28	0.63	1.72	-6.32E-03
**ND**	0.30	1.43	13.37	0.19	0.53	0.08	0.30	2.38E-03
**NE**	0.75	0.71	38.93	0.29	1.02	0.02	0.48	-2.80E-04
**NH**	12.52	14.50	39.45	0.20	1.56	5.99	1.35	0.13
**NJ**	46.97	48.36	94.59	1.56	2.28	2.69	1.33	0.71
**NM**	4.45	9.59	37.86	0.08	2.13	2.89	2.90	0.20
**NV**	2.03	2.14	10.92	0.05	0.36	0.34	0.82	-0.02
**NY**	58.21	74.64	78.18	1.46	2.06	26.70	1.15	0.56
**OH**	15.23	15.53	91.72	2.44	2.74	1.46	1.47	0.11
**OK**	9.53	6.93	74.83	0.47	1.40	0.49	1.47	0.01
**OR**	4.27	5.20	18.99	0.12	0.26	0.42	0.67	0.05
**PA**	18.01	18.37	82.67	2.68	2.41	5.56	1.62	0.22
**RI**	*55.17*	*162*	*2133*	*0.89*	*1.90*	*5.95*	*4.47*	*1.73*
**SC**	7.99	8.18	40.27	0.85	0.81	1.52	1.80	5.80E-03
**SD**	0.38	0.36	5.77	0.17	0.03	8.20	0.46	-2.69E-04
**TN**	10.53	11.29	61.81	1.25	1.58	2.99	1.97	-0.02
**TX**	12.96	12.93	125.35	0.52	1.87	1.12	1.81	0.07
**UT**	1.24	1.26	3.31	0.03	0.14	0.09	0.47	-7.01E-03
**VA**	30.05	31.38	133.40	3.77	3.56	5.67	4.88	0.19
**VT**	*24.88*	*7.75*	*17.97*	*0.44*	*3.23*	*1.12*	*8.87*	*0.83*
**WA**	9.76	10.31	14.08	0.45	0.53	0.35	0.48	0.25
**WI**	16.82	17.54	120.19	1.64	2.24	1.80	3.93	0.48
**WV**	13.91	14.76	78.83	2.38	2.37	2.20	2.00	0.01
**WY**	0.32	0.29	2.35	0.04	0.50	0.01	0.56	4.00E-05

a.

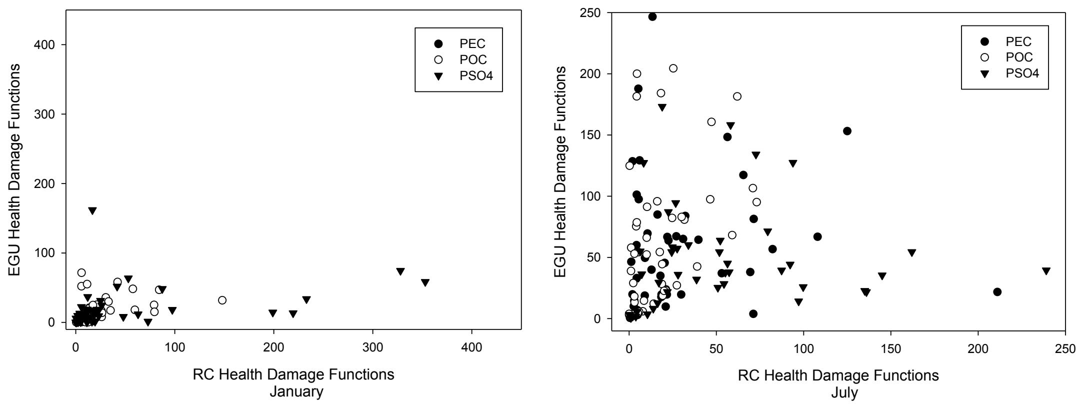

PEC
POC
PSO4
EGU Health Damage Functions
RC Health Damage Functions
January
July

b.

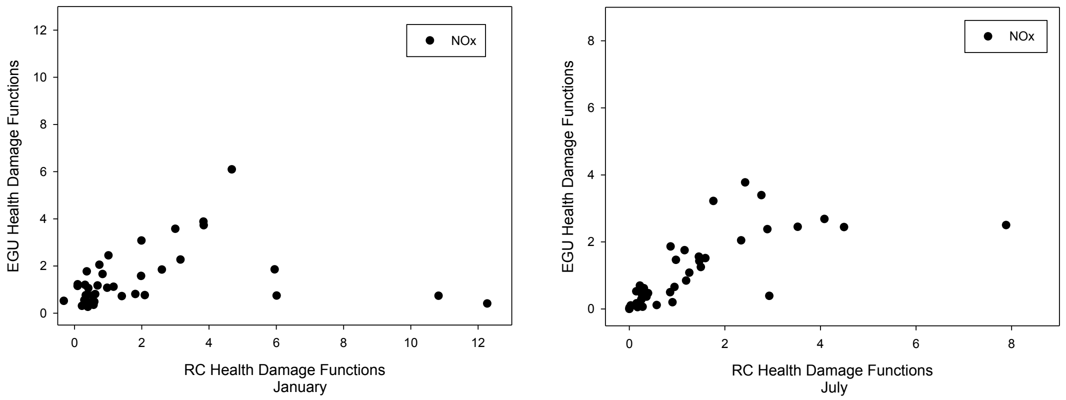

NOx
EGU Health Damage Functions
RC Health Damage Functions
January
July

c.

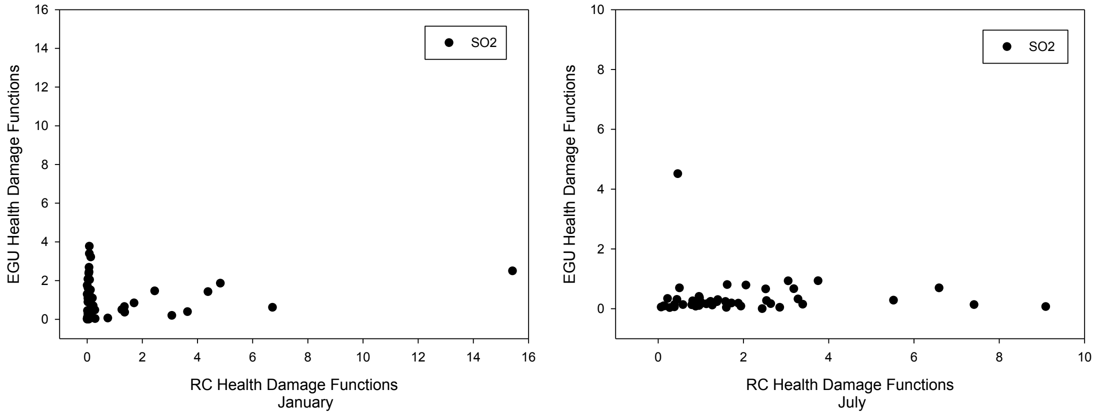

SO2
EGU Health Damage Functions
RC Health Damage Functions
January
July

d.

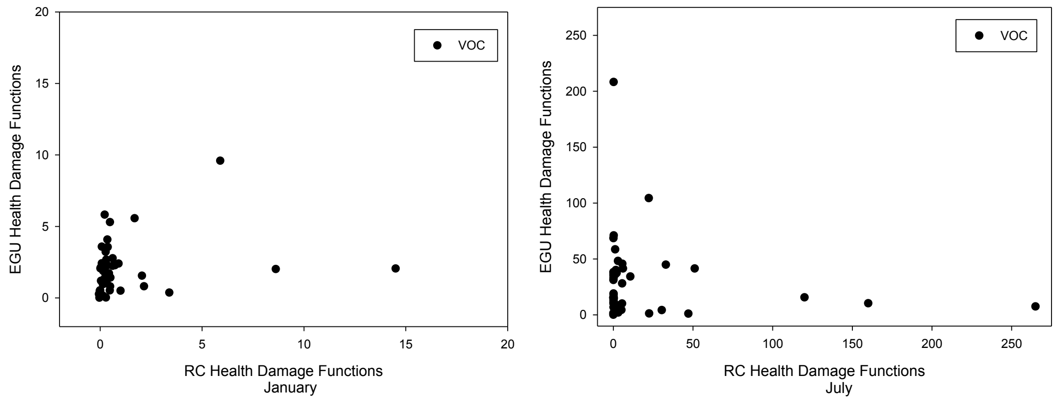

e.

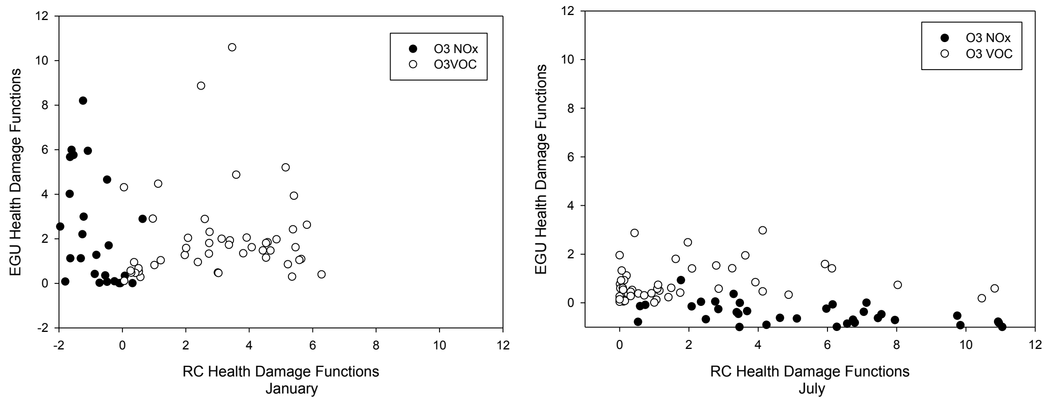

Figure S5. Scatterplots showing the relationship between health damage functions for RC (x-axis) and EGUs (y-axis) for individual source states related to primary $PM_{2.5}$ (panel a), NOx related to $PM_{2.5}$(panel b), SO_2 related to $PM_{2.5}$ (panel c), VOC related to $PM_{2.5}$ (panel d), and O_3 (panel e) for both January (left panel) and July (right panel).

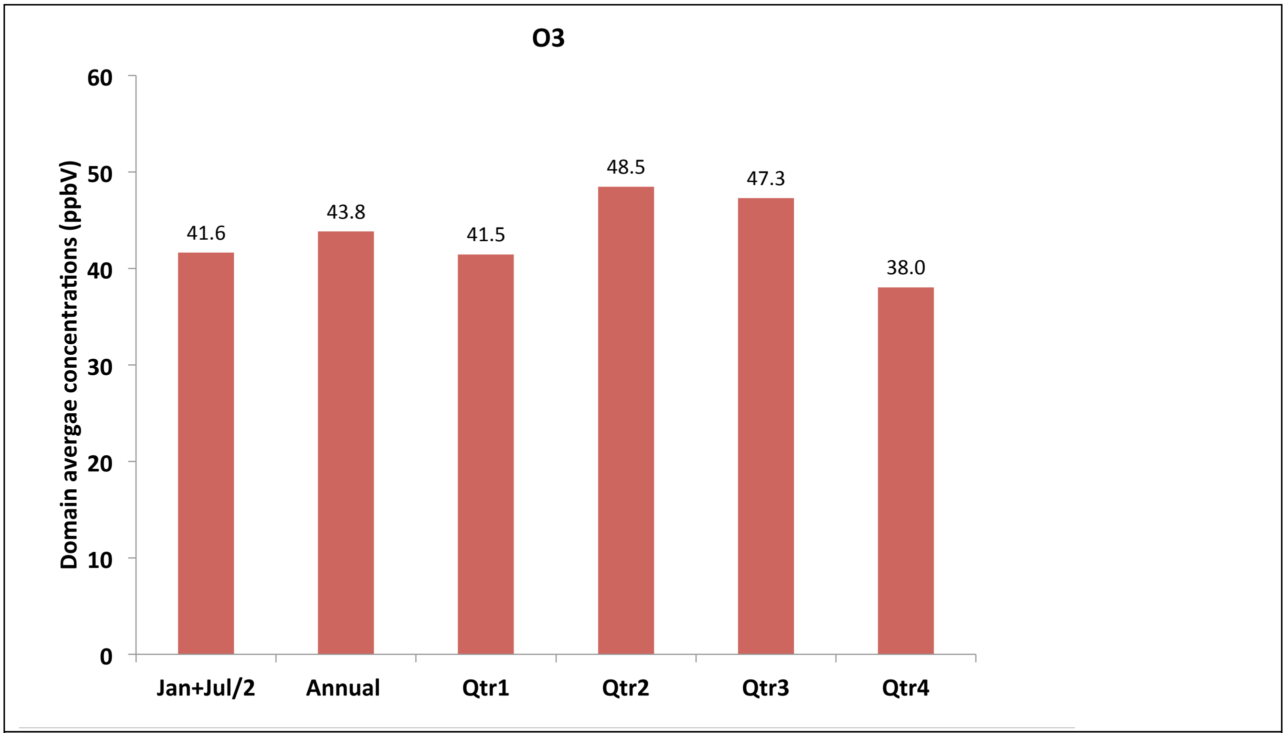

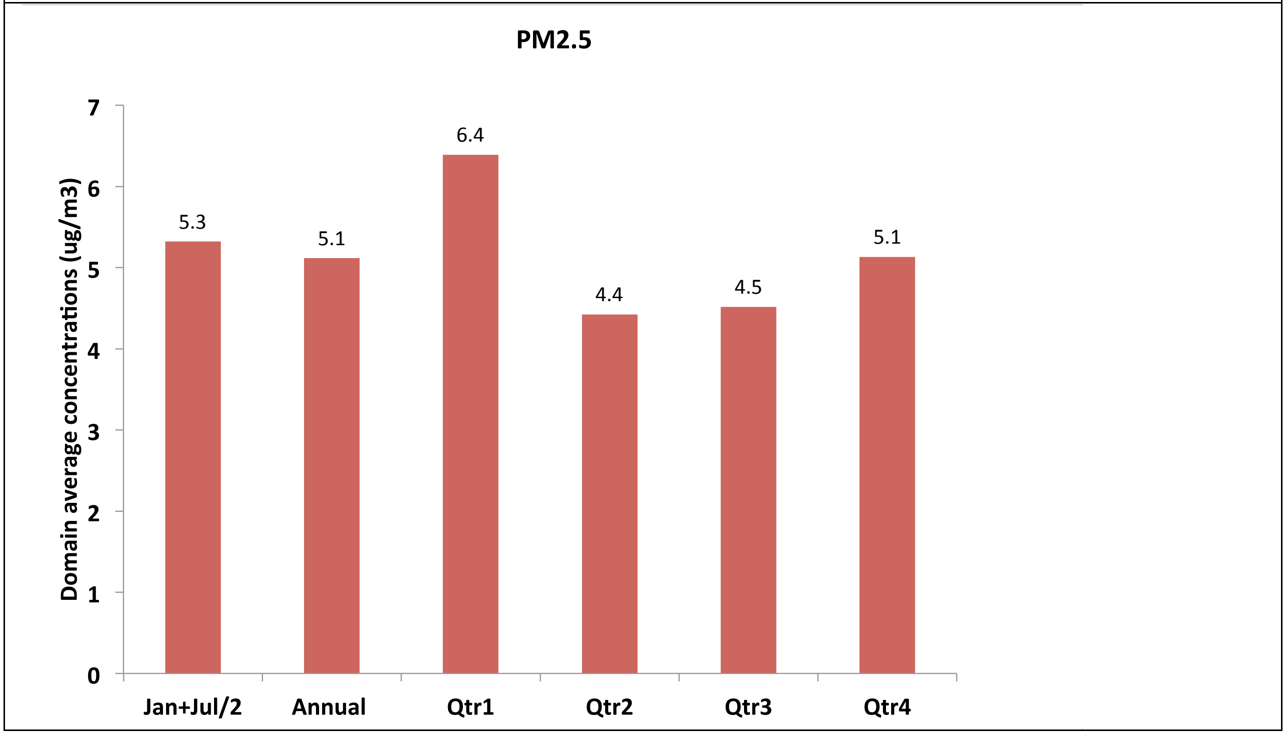

Figure S6. Domain-average O_3 and $PM_{2.5}$, compared for different periods of CMAQ simulations. (Qtr1 represents Jan-Mar, Qtr2 represents Apr-Jun, Qtr3 represents Jul-Sep and Qtr4 represents Oct-Dec).

References:

Ashok, A., I.H. Lee, S. Arunachalam, I. A. Waitz, S.H.L. Yim, R.H. Barrett, (2013). Development of a response surface model of aviation's air quality impacts in the US *Atmos. Environ.*, 77:445-452,

Boone, S., M. Omary, J.H. Bowden, S. Napelenok, S. Penn, J.I. Levy, S. Arunachalam (2016). Evaluation of air quality impacts from individual airports in the continental US using CMAQ DDM-3D/PM, In preparation.

Foley, K. M., Roselle, S. J., Appel, K. W., Bhave, P. V., Pleim, J. E., Otte, T. L., Mathur, R., Sarwar, G., Young, J. O., Gilliam, R. C., Nolte, C. G., Kelly, J. T., Gilliland, A. B., and Bash, J. O. (2010). Incremental testing of the Community Multiscale Air Quality (CMAQ) modeling system version 4.7, Geosci. Model Dev., 3, 205-226.

Foley, K.M. S. L. Napelenok, C. Jang, S. Phillips, B. J. Hubbell, C. M. Fulcher (2014). Two reduced form air quality modeling techniques for rapidly calculating pollutant mitigation potential across many sources, locations and precursor emission types, *Atmos. Environ.*,98:283-289.

Houyoux, M.R., Vukovich, J.M., Coats Jr., C.J., Wheeler, N.J.M., Kasibhatla, P.S., 2000. Emission inventory development and processing for the seasonal model for regional air quality (SMRAQ) project. *J. Geophys. Res.* 105 (D7), 9079 - 9090.

Lamarque, J.; Emmons, L.; Hess, P.; Kinnison, D.; Tilmes, S.; Vitt, F.; Heald, C.; Holland, E.; Lauritzen, P.; Neu, e. a. CAM-chem: description and evaluation of interactive atmospheric chemistry in CESM. *Geosci Model Dev* 2011, 4, 2199-2278.

Levy JI, Woo MK, Penn SL, Omary M, Tambouret Y, Kim CS, Arunachalam S. Carbon reductions and health co-benefits from US residential energy efficiency measures. 2016. *Environmental Research Letters* 11:3.

Rienecker, M. M.; Suarez, M. J.; Gelaro, R.; Todling, R.; Bacmeister, J.; Liu, E.; Bosilovich, M. G.; Schubert, S. D.; Takacs, L.; Kim, G.-K. MERRA: NASA's Modern-Era Retrospective Analysis for Research and Applications. *J Climate* 2011, 24.

Simon, H.; Baker, K. R.; Phillips, S. Compilation and interpretation of photochemical model performance statistics published between 2006 and 2012. *Atmos Environ* 2012, 61, 124 - 139.

Skamarock, W. C.; Klemp, J. B.; Dudhia, J.; Gill, D. O.; Barker, D. M.; Wang, W.; Powers, J. G. A description of the advanced research WRF version 2; 2005.

US Environmental Protection Agency (2011). Emissions Modeling for the Final Mercury and Air Toxics Standards Technical Support Document, EPA-454/R-11-011, Office of Air Quality Planning and standards, RTP, NC. December 2011.
